# MRI-guided photothermal/photodynamic immune activation combined with PD-1 inhibitor for the multimodal combination therapy of melanoma and metastases

**DOI:** 10.1093/rb/rbae019

**Published:** 2024-03-14

**Authors:** Changqiang Wu, Wei Chen, Shuang Yan, Jie Zhong, Liang Du, Chenwu Yang, Yu Pu, Yang Li, Jiafu Lin, Mei Zeng, Xiaoming Zhang

**Affiliations:** Medical Imaging Key Laboratory of Sichuan Province and School of Medical Imaging, North Sichuan Medical College, Nanchong 637000, P. R. China; Medical Imaging Key Laboratory of Sichuan Province and School of Medical Imaging, North Sichuan Medical College, Nanchong 637000, P. R. China; Medical Imaging Key Laboratory of Sichuan Province and School of Medical Imaging, North Sichuan Medical College, Nanchong 637000, P. R. China; Medical Imaging Key Laboratory of Sichuan Province and School of Medical Imaging, North Sichuan Medical College, Nanchong 637000, P. R. China; Medical Imaging Key Laboratory of Sichuan Province and School of Medical Imaging, North Sichuan Medical College, Nanchong 637000, P. R. China; Medical Imaging Key Laboratory of Sichuan Province and School of Medical Imaging, North Sichuan Medical College, Nanchong 637000, P. R. China; Medical Imaging Key Laboratory of Sichuan Province and School of Medical Imaging, North Sichuan Medical College, Nanchong 637000, P. R. China; Department of Radiology, Affiliated Hospital of North Sichuan Medical College, Nanchong 637000, P. R. China; Antibiotics Research and Re-evaluation Key Laboratory of Sichuan Province, Sichuan Industrial Institute of Antibiotics, School of Pharmacy, Chengdu University, Chengdu 610106, P. R. China; Institute of Basic Medicine and Forensic Medicine, North Sichuan Medical College and Institute of Rheumatology and Immunology, The Affiliated Hospital of North Sichuan Medical College, Nanchong 637000, P. R. China; Medical Imaging Key Laboratory of Sichuan Province and School of Medical Imaging, North Sichuan Medical College, Nanchong 637000, P. R. China

**Keywords:** photothermal therapy, photodynamic therapy, immunotherapy, magnetic resonance imaging, multimodal treatment

## Abstract

Non-invasive image-guided precise photothermal/photodynamic therapy (PTT/PDT) has been proven to be an effective local treatment modality but incompetent against metastases. Hence, the combination of local PTT/PDT and systemic immunotherapy would be a promising strategy for tumor eradication. Herein, a magnetic resonance imaging (MRI)-visualized PTT/PDT agent (SIDP NMs) was constructed, and the efficacy of its multimodal combination with a programmed cell death 1 (PD-1) inhibitor in the treatment of melanoma and metastases was studied. Due to the hydrophobic encapsulation of indocyanine green within the micellar core, SIDP NMs exhibited excellent photothermal/photodynamic properties and stability under an 808 nm near-infrared laser. *In vitro* cell experiments showed that SIDP NMs had a good killing effect. After incubating with B16-F10 cells for 24 h and irradiating with an 808-nm laser for 10 min, cell viability decreased significantly. Magnetic resonance imaging experiments in melanoma-bearing mice have shown that the dynamic distribution of SIDP NMs in tumor tissue could be monitored by *T*_2_WI and *T*_2_-MAP non-invasively due to the presence of superparamagnetic iron oxide nanocrystal in SIDP NMs. When the 808 nm laser was irradiated at the maximum focusing time point shown by MRI, the temperature of the tumor area rapidly increased from 32°C to 60.7°C in 5 min. In mouse melanoma ablation and distant tumor immunotherapy studies, SIDP NMs provided excellent MRI-guided PTT/PDT results and, when combined with PD-1 inhibitor, have great potential to cure primary tumors and eradicate metastases.

## Graphical abstract

**Figure:**
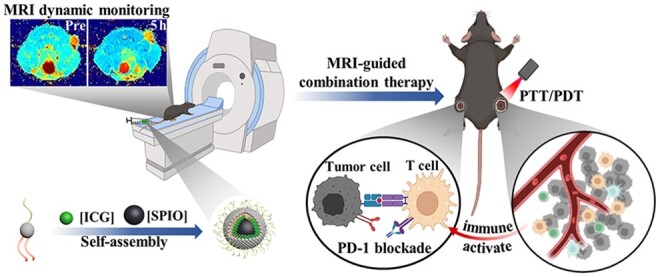

## Introduction

As non-invasive and effective cancer treatment modalities, PTT and PDT have gained a great deal of attention from the medical community [1], which destroy tumor cells by absorbing light energy and converting it into thermal energy or generating reactive oxygen species (ROS) [2, 3]. Furthermore, PTT and PDT trigger autoimmune responses by releasing cytokines, antigens and damage-associated molecular patterns (DAMPs) to recruit immune cells to attack tumor cells [4–7]. Chen *et al*. verified that hyperthermia in tumor caused by PTT could dilate the blood vessels, disintegrate the dense collagen fiber and reshape the tumor microenvironment (TME), which allows drugs and immune cells to enter the tumor easily [4]. The efficacy of PTT/PDT is mainly influenced by the photoconversion properties and the amount of photosensitizer accumulated in the tumor during laser irradiation. Despite traditional photosensitizers possessing great photoconversion capabilities, it is frequently difficult to obtain sufficient efficacy without the aid of imaging guidance. To date, the diagnosis-therapy-integrated photosensitive agent has drawn attention worldwide [8, 9]. Combining the non-invasive diagnostic modalities, such as computed tomography (CT), magnetic resonance imaging (MRI), positron emission tomography (PET), single photon emission tomography (SPECT), ultrasonic imaging (US), fluorescence imaging [10–14], makes it possible to non-invasively monitor the metabolism and distribution of therapeutic agents *in vivo* as well as determine the optimal time point to implement laser treatment. For example, Zhou et al. constructed Bi-Ag@PVP NPs for CT and photoacoustic imaging (PAI)-guided PTT/PDT, which significantly improved treatment efficacy [15]. However, the metabolic processes of bismuth and silver metal ions *in vivo* were not clear, and the safety of long-term application was difficult to guarantee. In addition, CT, PET and SPECT carry a risk of radiation damage during imaging. While PAI, fluorescence imaging and US could not clearly show the elaborate structure of the lesion, by contrast, MRI with ionizing radiation-free and outstanding spatial resolution to realize the accurate tracking of nanoparticles was considered an excellent option for visualizing treatments. Furthermore, iron was considered an essential element for physiological activities. Hence, iron-based MRI contrast agents are optimal candidates for imaging-guided therapy in terms of drug safety and imaging performance [16, 17]. Therefore, the development of low-toxicity and high-specificity photosensitizers with MRI visualization to realize precise and efficient therapy is a new goal for PTT/PDT.

At present, photosensitizers for tumor therapy mainly include gold nanoparticles, carbon quantum dots, semiconductor nanoparticles and organic dyes [7]. In addition, near-infrared-wavelength lasers have gained extraordinary interest in tumor therapy because of their good tissue penetration and low normal tissue absorption. Thus, indocyanine green (ICG), a US Food and Drug Administration-approved fluorescent dye for clinical application, has attracted much attention for its good near-infrared absorptivity and superior photothermal and photodynamic effects [18]. However, instability in the aqueous solution and blood due to severe photobleaching and binding to proteins in the blood limited ICG’s further application. To break these bottlenecks, various ICG-based photosensitizers have been developed. For example, the stability of ICG was improved by encapsulating poly-I-ornithine on the surface of ICG by solution casting approach [19–22]. In addition, improving the photothermal conversion mechanism of organic dyes to increase the generation of non-radiative heat is another key to the application of ICG for PTT/PDT. Among the many methods such as changing intramolecular motion, fluorescence resonance energy transfer, intra- and intermolecular charge transfer, and molecular stacking, encapsulation of amphiphilic molecules to fabricate nanoparticles not only addresses the drawbacks of ICG instability but also alters molecular stacking to improve non-radiative heat generation [23, 24]. Polydopamine (PDA) extracted from melanin, which is widely distributed in various parts of organisms, was widely utilized in bioimaging and tumor therapy because of its high chemical reactivity and easy functionalization properties. The presence of abundant chemically active functional groups (such as catechol, o-quinone, amine and imine) on the surface of PDA could be surface modified [25, 26], while some drawbacks of PDA still exist in biological applications involving cumbersome synthesis steps, uncontrollable structure and inferior photothermal conversion capacity [27]. For instance, NDs@PDA@ICG nanoparticles exhibit a large size (357.57 ± 8.63 nm), which makes it difficult to escape phagocytosis by the reticuloendothelial system *in vivo* and performs a mild photothermal capacity without the existence of ICG [28].

**Figure 1.**
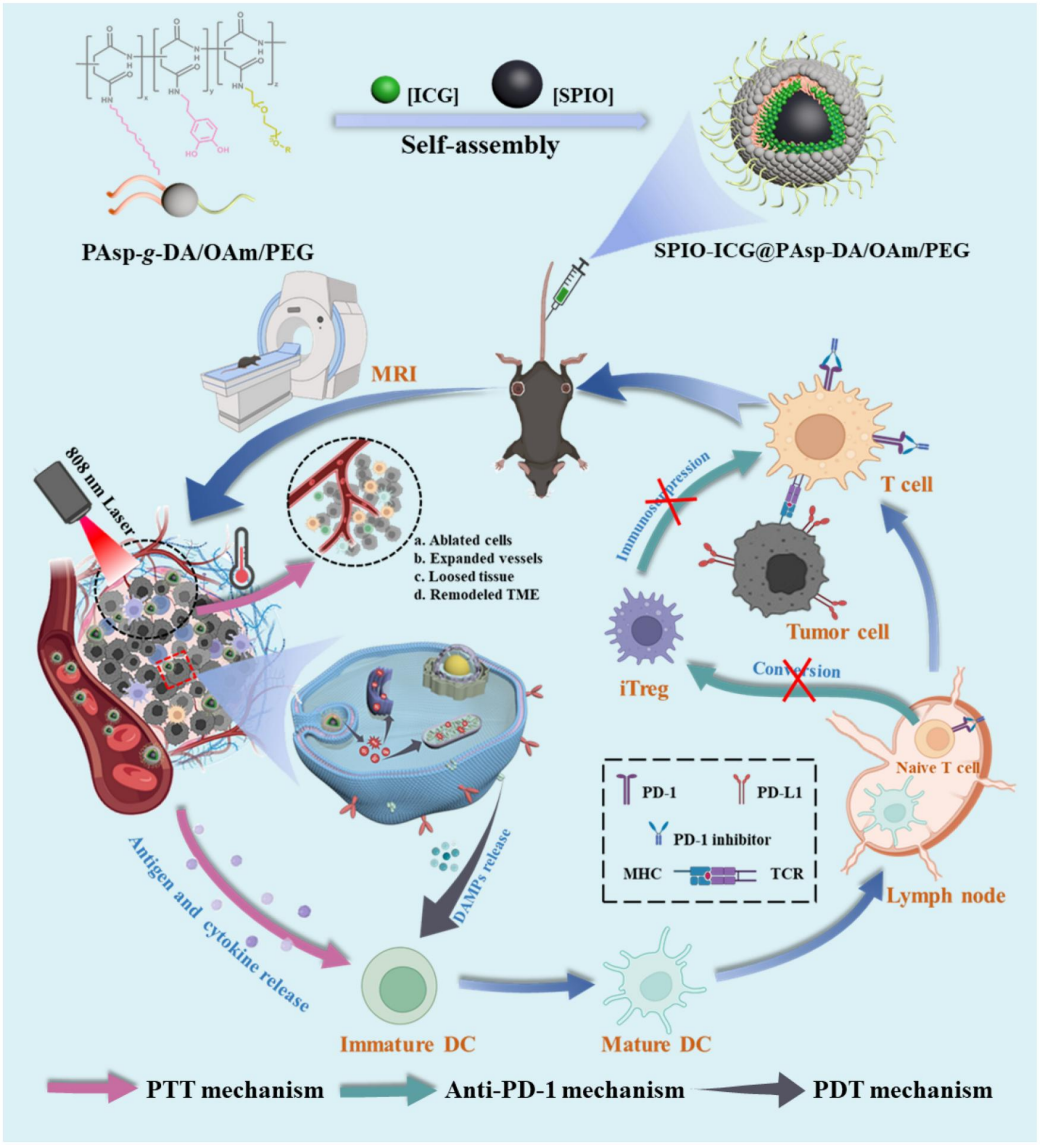
Schematic diagram illustrating the synthesis process of SIDP NMs and *in vivo* therapeutic mechanism in combination with PD-1 inhibitor.

Herein, versatile multimodal theranostic SPIO-ICG@PAsp-DA/OAm/PEG nanomicelles (SIDP NMs) were developed. As displayed in Figure 1, amphiphilic grafted polymer PAsp-*g*-DA/OAm/PEG was prepared by an acylation reaction, which utilized biocompatible polyaspartic acid as a framework, multivalently grafted dopamine to overcome the drawbacks of PDA synthesis and to obtain equivalent photothermal conversion ability. In addition, grafted hydrophobic oleylamine to form an amphiphilic polymer and polyethylene glycol grafted on the outer layer could improve the biocompatibility of nanomicelles to avoid phagocytosis by the reticuloendothelial system. Subsequently, small-size hydrophobic SPIO and ICG were encapsulated in nanomicelles to form safe and stable MRI-visualized PTT/PDT multifunctional therapeutic agents. In the TME, tumor cells were ablated effectively by SIDP NM-induced PTT/PDT. Meanwhile, the release of related antigens, cytokines and DAMPs induced by PTT/PDT promoted the maturation of DC cells, which could activate T cells to regress tumors [4, 6, 7]. Nevertheless, the numerous studies that have shown that PTT/PDT-induced immune activation was mild and insufficient against metastases [29, 30]. Thus, it is often combined with immunotherapy, which exerts anti-tumor effects by mobilizing autoimmune cells.

Conversely, tumor cells usually evade autoimmune attacks in many ways. Representatively, tumor cells communicated with programmed cell death 1 (PD-1) to inhibit T-cell proliferation and activation by blocking the Ras-Raf-MEK-ERK and PI3K-Akt signal pathways under the programmed cell death ligand 1 (PD-L1) umbrella, creating an immunosuppressive TME [31, 32]. By preventing the communication between tumor and T-cell immunological checkpoints, anti-PD-1/PD-L1 therapy could restore T-cell function and increase proliferation, enhancing the antineoplastic immune response [31–33]. Given the limited autoimmune ability and the degree of PD-1/PD-L1 expression, anti-PD-1/PD-L1 treatment was restricted to immunologically ‘cold’ tumors with few tumor-infiltration lymphocytes (TIL) [34–36]. Hence, considering the incompetence of PTT/PDT on metastases and the relatively low overall objective response rate of anti-PD-1, the combination of PTT/PDT and PD-1 blockade could efficiently ablate tumors and evoke robust autoimmunity to suppress metastases [37–39]. Herein, the potential of SIDP NMs combined with PD-1 inhibitor in MRI-guided PTT/PDT/immunology combination therapy of tumors and metastases was investigated.

## Materials and methods

### Synthesis of SPIO-ICG@PAsp-DA/OAm/PEG nanomicelles

ICG was dissolved in methanol and chloroform and mixed with SPIO and PAsp-*g*-DA/OAm/PEG dissolved in chloroform, then dropwise added into 10 ml of ultrapure water under ultrasonication. Then, the organic solvent was evaporated by rotary evaporation and dialyzed in ultrapure water for 24 h to remove unwrapped ICG (dialysis bag molecular weight cutoff: 10 kDa). Eventually, after filtering with a 0.22 μm syringe filter, SPIO-ICG@PAsp-DA/OAm/PEG nanomicelles were obtained and stored at 4°C in the dark for later use.

### *In vitro* cytotoxicity and hemolysis experiments

All cells mentioned in this study were purchased from Procell Life Science & Technology Co., Ltd. (Wuhan, Hubei, China). The cytotoxicity of SIDP NMs was evaluated by the Hep G2 cell line and the B16-F10 cell line. Hep G2 cells were incubated with different concentrations of SIDP NMs for 24 or 48 h and then stained with hoeches33342. Finally, the microplate reader (Thermos Scientifics, Varioskan LUX, USA) was employed to measure the fluorescent intensity of the cells at 455 nm (exciting wavelength 352 nm). The cell viability was calculated according to the formulas:

$$\mathrm{Cell}\;\mathrm{viability}=\frac{{IS}_{\mathrm{sample}}-{IS}_{\mathrm{blank}}}{{IS}_{\mathrm{control}}-{IS}_{\mathrm{blank}}}\times 100\%$$

Among them, the IS is the fluorescent intensity of the cell in each well at 455 nm.

For the hemolysis assay, red blood cells were isolated from healthy standard deviation (SD) rat blood and incubated with different concentrations of SIDP NMs at 37°C for 3 h followed by centrifugation to precipitate intact erythrocytes. PBS was used as the negative control and 0.1% Triton X-100 as the positive control. Then it was photographed, and the optical density values (OD) of the supernatant at 450 nm were measured with the microplate reader. To exclude the interference of SIDP NMs, the OD values of SIDP NM solutions with different Fe concentrations were also recorded, and the OD values of the corresponding SIDP NM solutions were deducted for each group of supernatants. The hemolysis rate was calculated by the formula:

$$\mathrm{Hemolysis}\;\mathrm{rate}=\frac{\mathrm{OD}-{\mathrm{OD}}_{\mathrm{PBS}}}{{\mathrm{OD}}_{\mathrm{Triton}\;X-100}-{\mathrm{OD}}_{\mathrm{PBS}}}\times 100\%$$

For the photothermal killing cell experiment, B16-F10 cells were incubated with different concentrations of SIDP NMs for 24 h, and excess SIDP NMs were discarded. Then, after 808 nm laser (1 W/cm^2^) irradiating 5 min (Blueprint, VCL-808nmM1-15.0W, China), stained with hoeches33342. Finally, the fluorescent intensity was measured and the cell viability was calculated.

### Magnetic resonance imaging

The *in vitro* MRI experiment with SIDP NMs solution was performed on a 3.0 T MRI device (GE, Discovery MR750, USA). SIDP NMs solution with different Fe concentrations (determined by inductively coupled plasma-mass spectrometry (ICP-MS, Perkin Elmer Nexion 350×, USA)) were placed in the chromatography bottle and fixed with 8% gelatin for the test. *T*_2_-weighted imaging (*T*_2_WI) was performed with a fast spin-echo sequence with the following parameters: TR = 3500 ms, TE = 130 ms, FOV = 200 × 70 mm, matrix = 384 × 256, thickness =1.0 mm, spacing = 0, echo chain length = 6, NEX = 4, receiving bandwidth = 31.25 kHz.

For *in vivo* tumor-bearing mice imaging, melanoma-bearing C57BL/6J mice were anesthetized by inhalation and then scanned using a 3.0 T MRI device equipped with a small animal-specific coil. Whole-body coronal *T*_2_-weighted imaging, axial *T*_2_-weighted imaging, and axial *T*_2_-MAP imaging of the tumor were performed at different time points after the tail vein injection of SIDP NMs. The scanning parameters are as follows:

*T*_2_-weighted fast spin-echo sequence (FSE): TR = 2500 ms, TE = 104 ms, FOV = 70 × 60 mm, matrix = 384 × 320, thickness = 1.0 mm, spacing = 0, echo chain length = 6, NEX = 3, receiving bandwidth = 50 kHz.

*T*_2_-MAP sequence: TR = 1200 ms, TE = 9-69 ms, FOV= 100 × 80 mm, matrix = 256 × 192, thickness = 2.0 mm, spacing = 0.6, echo chain length = 8, NEX = 2, receiving bandwidth = 62.5 kHz.

### Melanoma model and treatment strategies

The syngeneic transplantation mouse melanoma model was used for the PTT/PDT synergistic immunotherapy research [40]. Briefly, mouse B16-F10 melanoma cells (1 × 10^6^ cells) were injected subcutaneously in male C57BL/6J mice (6–8 weeks, 20 ± 5 g) right thigh and used for imaging or treatment when tumor reached 50–100 mm^3^ in volume. To evaluate the inhibitory effect of PTT/PDT in synergy with immunotherapy on primary and distant tumors, distant tumor was implanted in the left thigh of mice after 5 days of implantation of the primary tumor.

For anti-tumor therapy research, tumor-bearing mice were randomly divided into five groups (*n* = 4) to receive different treatments: (1) saline group (Saline, intravenous injection (i.v.)), (2) saline plus laser irradiation group (Saline + NIR), (3) anti-PD-1 treatment group (PD-1 inhibitor, 5 mg/kg, intraperitoneal injection (i.p.)) (Bioxcell, RMP1-14, USA), (4) SIDP NMs plus laser irradiation group (SIDP NMs + NIR, 5 mg/kg (i.v.)), (5) anti-PD-1 treatment plus SIDP NMs and laser irradiation group (anti-PD-1 + SIDP NMs + NIR). Laser-treated mice were irradiated with an 808 nm laser (0.7 W/cm^2^) for 5 min at 5 h after intravenous injection. Tumor volumes on both sides of the tumor-bearing mice and body weights were recorded daily until the end of treatment. The formula for calculating tumor volume is width × width × length × 0.5. Treatment was terminated when the tumor volume was greater than 1500 mm^3^ or the width of any side of the tumor exceeded 15 mm. Finally, the mice were sacrificed, and the main organs (liver, spleen, kidney, heart, lung) and tumor tissue were collected for hematoxylin and eosin (H&E) staining. In addition, calreticulin (CRT) expression and CD8^+^ T-cell population of tumor tissue were analyzed by fluorescent staining, and interleukin (IL)-12, IL-10, tumor necrosis factor-α (TNF-α), and transforming growth factor-β (TGF-β) levels were analyzed by enzyme-linked immunosorbent assay.

### Statistical analysis

IBM SPSS Statistics 25.0 software was used to perform statistical analysis. All data were represented as the mean ± SD. Significant differences were assessed by ANOVA. *P < 0.05 was considered significant; **P < 0.01 represents a highly significant difference between groups; and **P < 0.001 represents an extremely significant difference between groups.

More detailed experimental methods are available in the Supplementary information.

## Results and discussion

### Preparation and characterization of SIDP NMs

**Figure 2.**
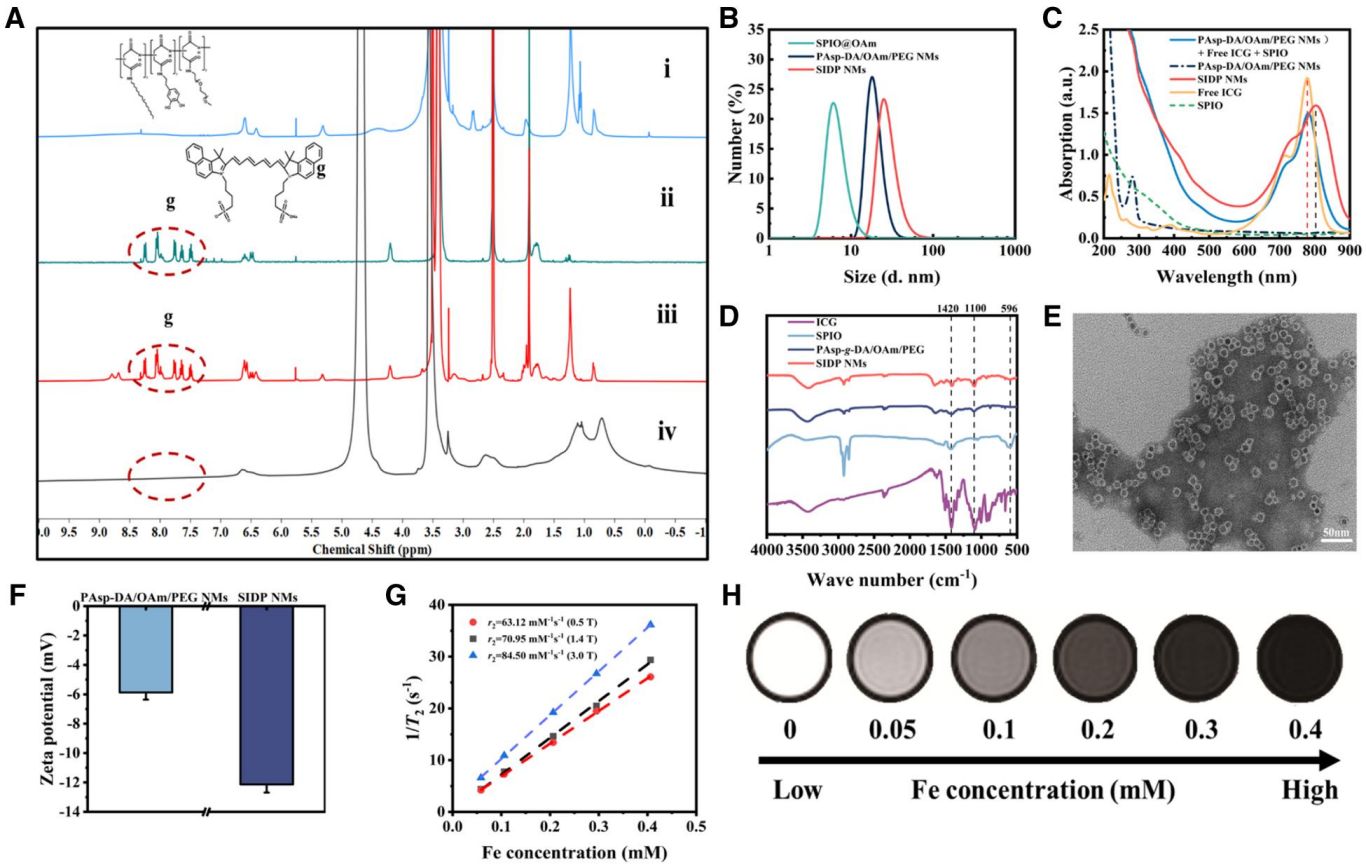
Characterization of SIDP NMs. (**A**) ^1^H NMR spectra of PAsp-*g*-DA/OAm/PEG in DMSO-*d*_6_ (i), ICG in DMSO-*d*_6_ (ii), ICG@PAsp-DA/OAm/PEG in DMSO-*d*_6_ (iii), ICG@PAsp-DA/OAm/PEG in D_2_O (iv). (**B**) Hydrodynamic size distribution of SPIO@OAm, PAsp-DA/OAm/PEG NMs, and SIDP NMs. (**C**) UV–Vis absorption spectra of SIDP NMs, PAsp-DA/OAm/PEG NMs, free ICG, SPIO solution, and mixed solution (PAsp-DA/OAm/PEG NMs + free ICG + SPIO). (**D**) FT-IR spectra of free ICG, SPIO, PAsp-*g*-DA/OAm/PEG, and SIDP NMs. (**E**) TEM image of SIDP NMs (negatively dyed by phosphotungstic acid). (**F**) Zeta potential of PAsp-DA/OAm/PEG NMs and SIDP NMs. (*n* = 3). (**G**) *T*_2_ relaxation efficiency-iron concentration curve of SIDP NMs solution under 0.5 T, 1.4 T, and 3.0 T main magnetic fields respectively. (**H**) *T*_2_-weighted image of SIDP NMs aqueous solution with different Fe concentrations under clinical 3.0 T MRI scanner (FSE sequence, TR = 3500 ms; TE = 130 ms).

The dopamine/oleylamine/polyethylene glycol-modified amphiphilic grafted polymer PAsp-*g*-DA/OAm/PEG was synthesized through three steps as shown in Supplementary Figure S1. The chemical structure of PAsp-*g*-DA/OAm/PEG was confirmed by ^1^H NMR spectra (Supplementary Figure S2). The peaks at 6.3–6.7 ppm and peaks at 3.5 ppm were assigned to the protons of phenyl (ph-H, c) in dopamine and the protons of PEG (–CH_2_–CH_2_–, d), respectively. The peaks around 0.8 and 1.2 ppm were attributed to the terminal methyl protons (–CH_3_, a) and methylene protons (–CH_2_–, b) of oleylamine respectively. Peaks at 4.4, 1.9 and 2.9 ppm assigned the protons of methylene and methyne (–COCH–, e; –COCH_2_–, f) in the polyaspartate skeleton. The ^1^H NMR spectra of PAsp-*g*-DA/OAm/PEG revealed that dopamine, oleylamine and polyethylene glycol were successfully grafted on the side groups of polyaspartic acid. ICG was encapsulated in nanomicelles by the self-assembly of amphiphilic PAsp-*g*-DA/OAm/PEG. The ^1^H NMR spectra of ICG@PAsp-DA/OAm/PEG in dimethyl sulfoxide-*d*_6_ (DMSO-*d*_6_) and deuterium oxide (D_2_O) were used to verify the location of ICG in the hydrophobic nucleus of the PAsp-DA/OAm/PEG nanomicelles. In DMSO-*d*_6_, ICG and PAsp-*g*-DA/OAm/PEG will be dissolved into dispersed molecules, and the characteristic peaks of dopamine (peak c), oleylamine (peaks a and b), mPEG_2000_ (peak d) and the naphthalene proton of ICG (peak g) could be seen in the ^1^H NMR spectrum (Figure 2A and Supplementary Figure S2). In D_2_O, the ICG was encapsulated in the hydrophobic core of the micelle; thus, the naphthalene proton peak (peak g) was not detected in the ^1^H NMR spectrum.

As a promising MRI contrast agent, SPIO could be combined with various carriers to achieve non-invasive dynamic monitoring *in vivo*. To make the nanosystem equipped with MRI-visualized capability, hydrophobic SPIO was simultaneously encapsulated in PAsp-DA/OAm/PEG nanomicelles, and the SIDP NMs were obtained. To obtain a desirable photosensitizers with superior relaxation efficiency, we synthesized nanomicelles with different ratios and evaluated their physicochemical properties by measuring size, absorption at 808 nm, and relaxation efficiency. According to the measured results (Supplementary Figures S3–S5 and Table S1), the absorption effect and size of SIDP NMs are optimal when the proportion of each component in the content is m_(Polymer)_:m_(Contents)_ = 3:1 and m_(SPIO)_:m_(ICG)_ = 2:1 while ensuring the imaging effect. This composition of micelles was used in all subsequent experiments.

The average hydrodynamic diameter of SIDP NMs was 24.5 ± 1.0 nm with a narrow particle size distribution (Polydispersity index (PDI) = 0.397) measured by dynamic light scattering (DLS) (Figure 2B), which was slightly increased compared to hydrophobic SPIO nanocrystals (6.4 ± 0.4 nm, PDI = 0.211) and the PAsp-DA/OAm/PEG micelles without loaded SPIO and ICG (16 ± 0.5 nm, PDI = 0.394). After ICG encapsulated inside the micelles, the characteristic peak at 779 nm of the ICG monomer on the UV–Vis spectrum was red-shifted to 803 nm (Figure 2C), which could be caused by the Π–Π stacking of ICG molecules inside the micelles [13]. In contrast, no shift of characteristic peak was observed for the ICG free from the nanomicelles in the mixed solution. According to the Fourier transform infrared (FT-IR) spectrum depicted in Figure 2D, the characteristic peaks at 596, 1100 and 1420 cm^−1^ were, respectively, ascribed to SPIO, PEG and ICG of SIDP NMs, confirming the existence of SPIO and ICG within the micelles. As displayed in Figure 2E and Supplementary Figure S6, transmission electron microscopy (TEM) images showed that both SPIO and SIDP NMs had a homogeneous microsphere morphology with an average size of 6.6 ± 0.1 nm for SPIO and a slight increase in the size of 10.6 ± 1.8 nm after polymer wrapping, which was smaller than hydrodynamic diameter and may be related to the dehydration that occurred during the preparation of TEM samples. The zeta potential was −5.9 mV for PAsp-DA/OAm/PEG NMs (Figure 2F). Importantly, the zeta potential of SIDP NMs was −12.1 mV after loading SPIO and ICG. The surface negative potential of SIDP NMs was conducive to avoid charge-dependent adsorption of serum proteins and increase the residence time of nanomicelles *in vivo* [41]. To further verify the possibility of SIDP NMs in MRI application, *T*_1_ and *T*_2_ relaxivity (*r*_1_ and *r*_2_) of SIDP NMs with different Fe concentrations at 0.5 T, 1.4 T and 3.0 T was measured, and results are presented in Figure 2G and Supplementary Figure S7. The relaxivity of SIDP NMs solutions under a 3.0 T was calculated as *r*_1_ = 1.11 Fe mM^−1^ s ^−1^, *r*_2_ = 84.50 Fe mM^−1^ s^−1^, and *r*_2/_*r*_1_ = 76.12, which were employed as *T*_2_ contrast agents. Furthermore, *T*_2_WI was performed on SIDP NMs aqueous solutions with different Fe concentrations under 3.0 T MRI scanner (Figure 2H), demonstrating the potential of SIDP NMs for *in vivo* MRI visualization. The above results implied that SIDP NMs had great potential for *in vivo* applications.

The short circulating half-life of ICG was mainly due to the structural properties of ICG, which were easily bound to plasma proteins and low-density lipoproteins in the blood and thus rapidly metabolized by the liver, limiting the application of ICG in oncology therapy [42]. These obstacles might be resolved by wrapping ICG in PAsp-DA/OAm/PEG nanomicelles to form a stabilized nanoplatform and prolong the *in vivo* circulation period of ICG. The encapsulation efficiency and loading capacity of ICG in SIDP NMs were calculated at 54% and 6% by the UV–Vis absorption spectroscopy quantitative curve, respectively (Supplementary Figure S8A and B). To evaluate the stability of ICG-incorporated nanoparticles, the hydrodynamic size of SIDP NMs in aqueous solution was monitored for 7 days and we did not find any significant changes (Supplementary Figure S9A). Evidently, the stability of the blood is even more important for the *in vivo* application. Thus, 20% (v/v) fetal bovine serum was incubated with SIDP NMs at 37°C for 24 h, and no increase in hydrodynamic size was observed by DLS measurements (Supplementary Figure S9B), indicating that ICG was encapsulated in micelles blocking binding to proteins and favoring increased circulation time in the vascular. Collectively, the aforementioned results of SIDP NMs laid an important foundation for subsequent *in vivo T*_2_-weighted imaging and therapeutic studies in mice.

### *In vitro* photothermal properties of SIDP NMs

**Figure 3.**
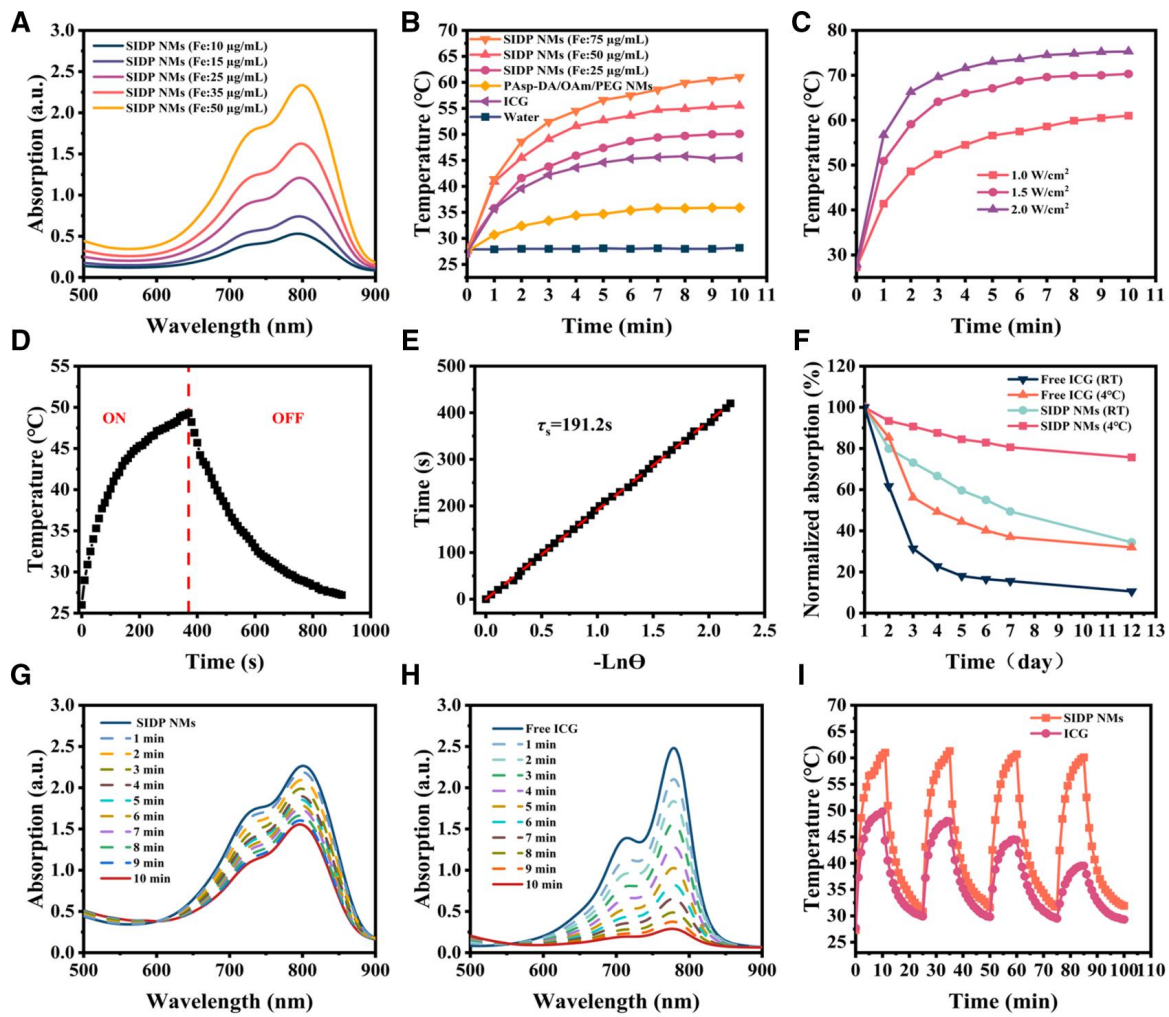
*In vitro* photothermal properties of SIDP NMs. (**A**) Near-infrared absorption spectra of SIDP NMs solution with varied Fe concentrations. (**B**) Temperature variation curves of SIDP NMs solution with different Fe concentrations, PAsp-DA/OAm/PEG NMs, ICG solution (concentration comparable to 75 µg/ml Fe SIDP NMs), and water under 808 nm laser irradiation (1 W/cm^2^). (**C**) Temperature change curves of SIDP NMs solution (Fe concentration: 75 µg/ml) under 808 nm laser irradiation with different optical power density. (**D**) Heating and cooling curves of SIDP NMs solution (Fe concentration: 30 µg/ml) under 808 nm (1 W/cm^2^) laser irradiation on and off. The laser was turned off after 370 s. (**E**) Linear correlation of the cooling time versus negative natural logarithm of driving force temperatures. The time constant is calculated as *τ_s_* = 191.2 s. (**F**) Time-dependent variation of normalized characteristic absorption values for free ICG (779 nm) and SIDP NMs (803 nm) solution storage at different temperatures. The UV–Vis absorption spectra of (**G**) SIDP NMs solution and (**H**) free ICG after 808 nm laser irradiation (1 W/cm^2^). (**I**) Temperature change curves of SIDP NMs and ICG aqueous solution after four on/off cycles of 808 nm laser irradiation (1 W/cm^2^).

A therapeutic agent with promising photothermal conversion properties is one of the most important factors in determining intrinsic therapeutic performance. To investigate the photothermal properties of SIDP NMs, the UV–Vis absorption spectra of SIDP NMs solutions containing different Fe concentrations were examined. As shown in Figure 3A, SIDP NMs exhibited a wide range of absorption in the near-infrared band, and the absorption increased with Fe concentration. Inspired by strong near-infrared absorption, we investigated the photothermal conversion properties induced by an 808 nm laser. The photothermal temperature changes and the thermograms of SIDP NMs with different Fe concentrations under 808 nm laser (1 W/cm^2^) irradiation are shown in Figure 3B and Supplementary Figure S10. The temperature of the SIDP NMs solution at 75 µg/ml increased from 28°C to 61°C after 10 min of laser irradiation, which was enough to instantly destroy tumor cells [43], whereas equivalent concentrations of ICG only elevated 18.3°C and PAsp-DA/OAm/PEG micelles merely increased 8.4°C with the presence of dopamine when no obvious temperature change was observed in water. With the laser power varying from 1 to 2 W/cm^2^, the temperature was monitored with 75 µg/ml of SIDP NMs solution (Figure 3C). The photothermal conversion efficiency of SIDP NMs was calculated at 14.8% by fitting curves during cooling (Figure 3D and E), which was comparable to free ICG (15.5%, Supplementary Figure S11). These results demonstrated that ICG and dopamine in SIDP NMs cooperatively exert excellent and stable photothermal conversion effects.

The unsatisfactory stability of the aqueous solution and blood is the major factor impeding the application of ICG with excellent photothermal and photodynamic conversion capacity [44]. Thus, to explore the stability of ICG encapsulated in SIDP NMs solution, we monitor the characteristic absorption peak changes of SIDP NMs and free ICG solution with different temperature conditions over 12 days (Figure 3F). Whether stored at 4°C or room temperature, the absorption peak of free ICG declined rapidly within 7 days, whereas SIDP NMs decreased slowly. After being stored at 4°C for 12 days, the absorption of SIDP NMs barely decreased by 24.4%, revealing that wrapping ICG inside micelles significantly increased the stability of ICG in aqueous solutions. Moreover, to further confirm the photothermal stability of SIDP NMs, the absorption spectra of the free ICG and SIDP NMs solutions were measured after 1 W/cm^2^ laser irradiation (Figure 3G and H). The results showed that the absorption of free ICG was almost entirely degraded, while the SIDP NMs declined slightly after 10 min. Meanwhile, the temperature profile after four cycles of laser on/off was recorded and also exhibited no obvious changes in the highest temperature (Figure 3I), while the opposite was observed for ICG. The above results indicated that SIDP NMs had superior photothermal properties and stability *in vitro*, which was conducive to further applications *in vivo*.

### *In vitro* photodynamic properties of SIDP NMs

**Figure 4.**
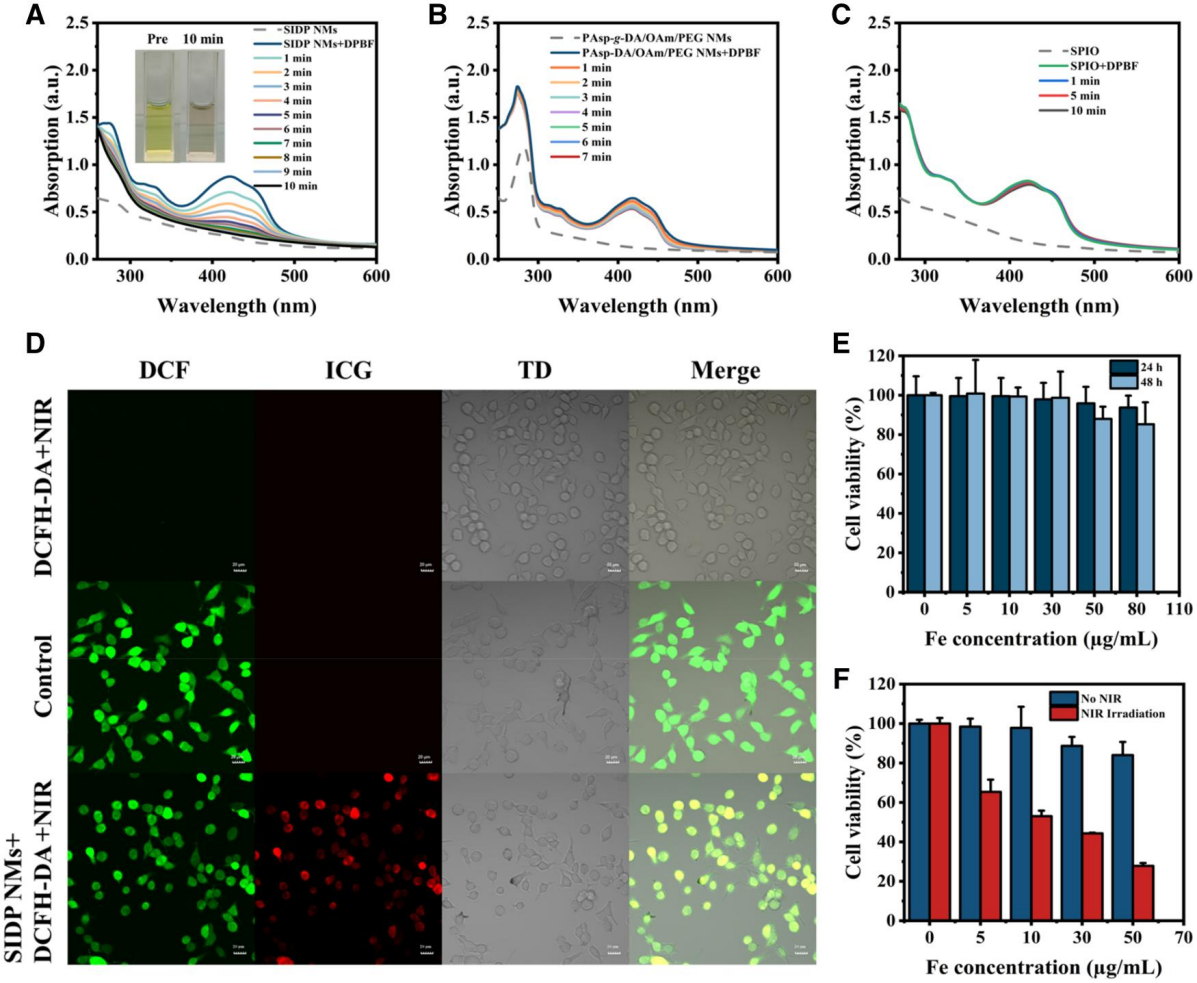
*In vitro* photodynamic properties and biocompatibility of SIDP NMs. Absorption spectra of DPBF mixed with (**A**) SIDP NMs solution, (**B**) PAsp-DA/OAm/PEG NMs and (**C**) SPIO under 808 nm laser irradiation (1 W/cm^2^) for different time. (**D**) Fluorescence images of B16-F10 cells treated with DCFH-DA + NIR, positive stimulation and SIDP NMs + DCFH-DA + NIR, respectively; scale bar, 20 μm. (**E**) Cell viability of hep G2 cells incubated with SIDP NMs for 24 h and 48 h (*n* = 3). (**F**) Cell viability of B16-F10 cells cultured with SIDP NMs after 24 h with and without 808 nm laser irradiation (1 W/cm^2^, 10 min) (*n* = 3).

For evaluating the photodynamic properties of SIDP NMs, 1,4-diphenyl-2,3-benzofuran (DPBF) and 2′,7′-dichlorofluorescin diacetate (DCFH-DA) probes were employed to estimate the production of singlet oxygen (^1^O_2_) and ROS by SIDP NMs in solution and tumor cells, respectively. The reaction of ^1^O_2_ with DPBF in the system led to the loss of the extended Π-electron system and its characteristic spectral (417 nm), resulting in the generation of the endoperoxide 1,2-dibenzoylbenzene [45]. Additionally, laser irradiation may change the absorption intensity of SIDP NMs at 417 nm because of the instability of ICG. To more accurately detect ^1^O_2_ with the DPBF probe, the absorption at 417 nm was also monitored after 808 nm laser (1 W/cm^2^) irradiation. The findings demonstrated that SIDP NMs showed almost no change at 417 nm after laser irradiation (808 nm, 1 W/cm^2^) without DPBF, ruling out the influence of the SIDP NMs themselves (Supplementary Figure S12). Subsequently, SIDP NMs (10 µg/ml, 2 ml) were mixed with a DPBF probe (2 mg/ml, 10 µl) to focus on the ^1^O_2_ production at 808 nm laser (1 W/cm^2^) irradiation by using a UV–Vis spectrophotometer. As shown in Figure 4A, DPBF was almost completely depleted within 10 min of laser irradiation, suggesting that SIDP NMs had a powerful ^1^O_2_ production capacity under a laser. To further clarify the photodynamic properties of SIDP NMs, the production of ^1^O_2_ from SPIO solutions and PAsp-DA/OAm/PEG micelles was also examined (Figure 4B and C). In the PAsp-DA/OAm/PEG micelle, the typical absorption of DPBF was significantly decreased by laser irradiation due to the existence of dopamine with photodynamic capacity, whereas there was almost no effect in the SPIO solution. Meanwhile, the absorption spectrum of the DPBF solution without SIDP NMs was still maintained at 417 nm as a control (Supplementary Figure S13). The above results illustrated that ICG and dopamine in SIDP NMs could synergistically exhibit superior photodynamic properties *in vitro*, presenting good potential in tumor photodynamic therapy. Next, we explored SIDP NMs-mediated cellular oxidative stress by characterizing the change in the ROS level in B16-F10 cells using the DCFH-DA probe [46]. As revealed in Figure 4D, the positive control group (control) and the experimental group (SIDP NMs + DCFH-DA + NIR) equally displayed strong green fluorescence. Additionally, the red fluorescence of the cells appeared in the experimental group mainly because of the presence of ICG in SIDP NMs. While no ROS or ICG were observed in the negative control group (DCFH-DA + NIR), these results meant that SIDP NMs also exhibited good photodynamic properties in cells.

### *In vitro* biocompatibility and cytotoxicity of SIDP NMs

To evaluate the biocompatibility of SIDP NMs, cytotoxicity, and hemolysis experiments were performed. As shown in Figure 4E, no cytotoxicity was observed after incubating hepatoma cells (Hep G2) with SIDP NMs, even though the Fe concentration was up to 80 µg/ml for 48 h, and the cell viability was still above 85%. Moreover, no obvious hemolysis phenomenon was observed after incubation of erythrocytes with SIDP NMs even at a Fe concentration of 150 µg/ml (Supplementary Figure S14), indicating the superior safety of SIDP NMs. The color gradually deepened with increasing Fe concentration due to the color of SIDP NMs itself, and no increase in OD at 450 nm of the supernatant was observed after subtracting the absorbance of SIDP NMs. In addition, the photothermal kill ability of SIDP NMs on mouse melanoma cells (B16-F10) was similarly verified. There was a slight decrease with increasing Fe concentration without 808 nm laser irradiation after incubating the B16-F10 cells and SIDP NMs, while the cell viability decreased significantly with increasing concentration with 808 nm laser irradiation (1 W/cm^2^) for 10 min, exhibiting outstanding photothermal ability of SIDP NMs (Figure 4F). In addition, we further explored the interactive role of SIDP NMs and B16-F10 cells by using the fluorescence properties of ICG (Supplementary Figure S15) and showed that SIDP NMs were phagocytosed by B16-F10 cells and presented in the cytoplasm after incubation for 6 h. These results demonstrated that SIDP NMs had excellent biocompatibility and exhibited great PDT potential for tumors.

### Dynamic analysis of SIDP NMs accumulation in tumors by MRI

**Figure 5.**
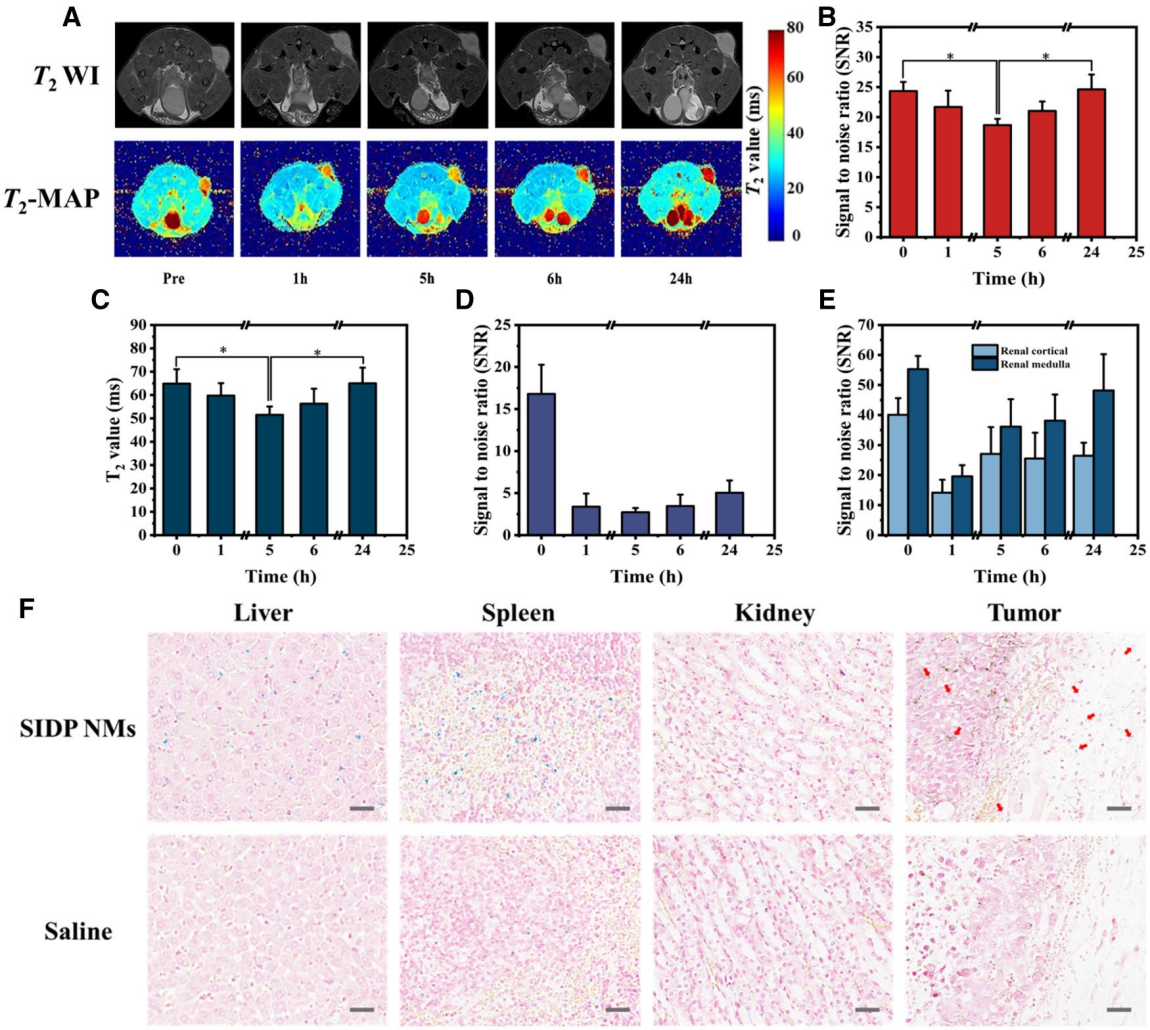
*In vivo* metabolic distribution of SIDP NMs in melanoma-bearing mice monitored by MRI. (**A**) *In vivo T*_2_-weighted images (TR = 3500 ms, TE = 130 ms) and *T*_2_-MAP images (TR = 1200 ms, TE = 9–69 ms) of tumor-bearing mice at different time points after intravenous injection of SIDP NMs (5 mg/kg). (**B**) Signal-to-noise (SNR) ratio and (**C**) *T*_2_ values change at different time points of tumor sites after intravenous injection of SIDP NMs measured by (A) (*n* = 4). SNR ratio changes in (**D**) liver and (**E**) kidney of tumor-bearing mice at different time points after intravenous injection of SIDP NMs measured by *T*_2_-weighted images (*n* = 4). (**F**) Prussian staining of liver, spleen, kidney and tumor tissue sections from melanoma-bearing mice after injection of SIDP NMs and saline 5 h; scale bar, 50 μm.

The existence of SPIO in nanomicelles imparted MRI *T*_2_ contrast properties to SIDP NMs, which might guide the photosensitizer for precise PTT/PDT *in vivo*. MRI *T*_2_WI and *T*_2_-MAP were performed on melanoma-bearing mice to analyze the metabolic distribution of SIDP NMs *in vivo*. As displayed in Figure 5A–C, a maximum decline in *T*_2_ signal and *T*_2_ values in the tumor was evident after intravenous injection of SIDP NMs for 5 h, reflecting the maximum accumulation of SIDP NMs in the tumor region at this time point, which was the optimal occasion for laser treatment. The content of nanoparticles accumulated in tumors partly depends on the design of the nanomaterial itself. Although coated with biocompatible PEG, the nanoparticles could not escape being phagocytosed by phagocytes in the reticuloendothelial system of the liver and spleen *in vivo* [47], and thus the liver displayed a diminished signal on MRI (Figure 5D and Supplementary Figure S15A). After being metabolized in hepatic Kupffer cells, SPIO was demonstrated to be deposited in the mononuclear phagocytic system to form complexes with ferritin and iron-containing heme and therefore not cause liver damage due to iron overload [48]. Interestingly, the *T*_2_ signal in the kidney also declined (Figure 5E and Supplementary Figure S15B). It is presumed that a fraction of small-size SIDP NMs could pass through the glomerular filtration barrier (GFB) and then be excreted through the kidney [49]. On the other hand, nanomicelles that were soft materials could be ‘squeezed’ through the pores of GFB [50]. The distribution of SIDP NMs in major metabolic organs and tumors after injection for 5 h was also confirmed by Prussian blue staining. As depicted in Figure 5F, the large number of blue particles in the liver and spleen reflected the fact that nanoparticles are mainly phagocytosed by the reticuloendothelial system of the liver and spleen. Moreover, blue particles were observed in tumors of mice injected with SIDP NMs compared to the saline group, which validated the change in MRI signal induced by SIDP NMs *in vivo*. These results demonstrated that the metabolism and distribution of SIDP NMs *in vivo* could be visualized by MRI and were consistent with Prussian blue staining, which was sufficient to guide *in vivo* precise PTT/PDT.

### MRI-guided photothermal/photodynamic therapy and immune activation combined with PD-1 inhibitor for melanoma

**Figure 6.**
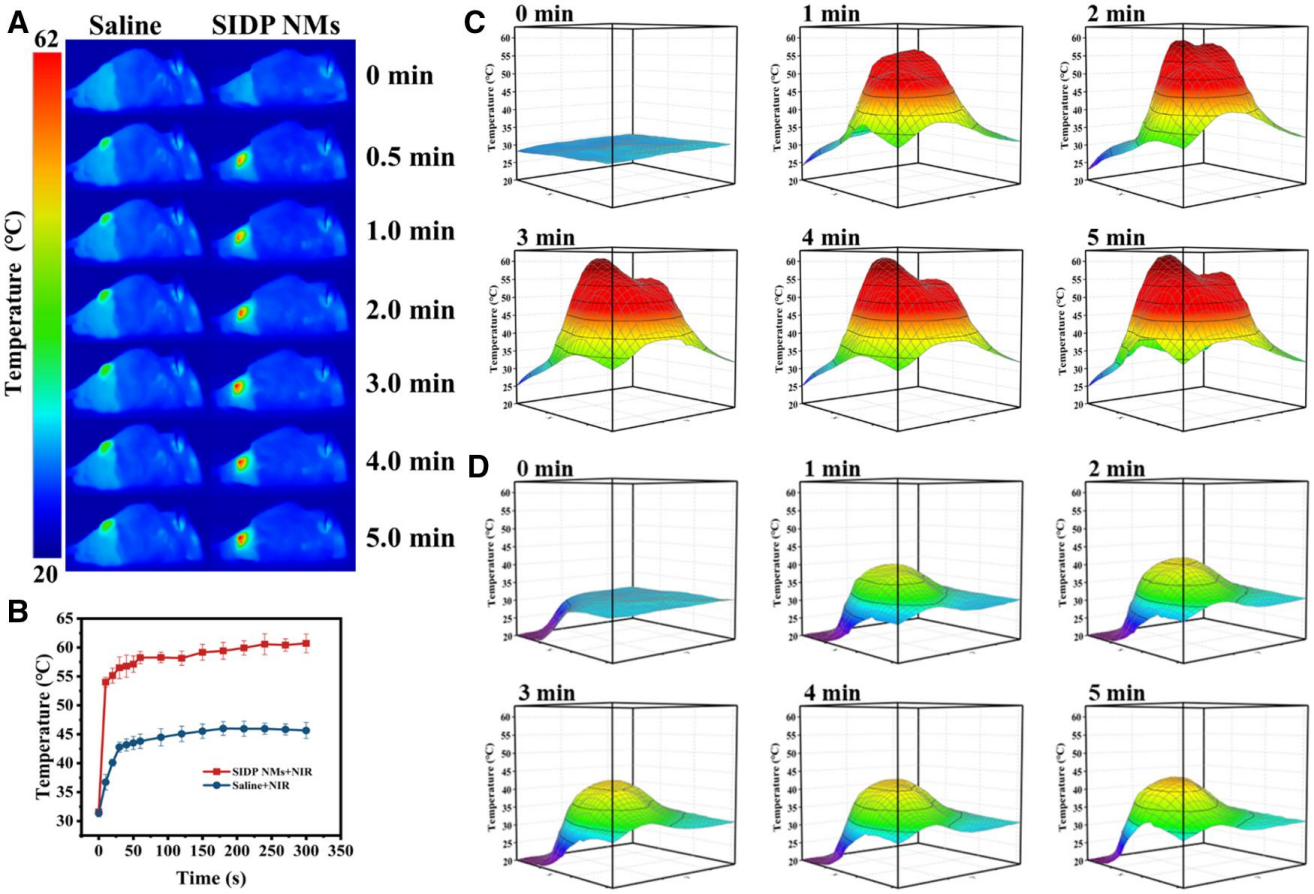
*In vivo* photothermal performance of SIDP NMs. (**A**) Infrared thermography and (**B**) temperature change curves of the tumor within 5 min under 808 nm laser irradiation (0.7 W/cm^2^) after intravenous injection of saline and SIDP NMs. Three-dimensional temperature maps of tumor in mice after injected with (**C**) saline and (**D**) SIDP NMs irradiated by 808 nm laser at different time (0.7 W/cm^2^).

Encouraged by the photo-induced cytotoxicity of SIDP NMs *in vitro* and MRI-guided *in vivo*, the *in vivo* SIDP NMs-triggered PTT/PDT and combined immunotherapy efficacy were investigated. Due to the limited penetration ability of the 808 nm laser, it is mostly used for superficial tumors, such as breast cancer and skin cancer, or deep tumors, such as pancreatic cancer, through interventional therapy [51–55]. Therefore, the syngeneic transplantation mouse melanoma model was utilized for *in vivo* efficacy studies. The laser treatment was carried out after administration of SIDP NMs for 5 h when the micelles were maximally aggregated in the tumor under MRI guidance. The maximum temperature at the tumor region in SIDP NMs + NIR treated mice increased over time from 32°C to 60.7°C under 0.7 W/cm^2^ 808-nm laser irradiation, which was sufficient to cause cell necrosis or apoptosis in a short period [56]. In contrast, mice-administrated saline + NIR showed a slight rise in the maximum temperature at the tumor area only from 30°C to 45.6°C due to the properties of melanoma itself (Figure 6A and B). At a temperature below 48°C, the proteins would aggregate but are insufficient to prevent the expansion of the tumor alone [2, 57]. In addition, to exclude the effect of C57BL/6J mouse skin on warming, skin heating under laser irradiation was monitored, which confirmed that the skin was only heated slightly (Supplementary Figure S17). The infrared thermography of mice and the three-dimensional temperature map of the tumor region confirmed that laser irradiation did not cause a uniform temperature increase in the tumor area (Figure 6C and D). Only the center of the tumor reached the temperature that was capable of rapidly ablating tumor cells in the SIDP NMs + NIR group, and tumor margins formed a temperature gradient where cells did not receive immediately fatal thermal doses but were exposed to temperatures over 45°C. It has been established that mild photothermal therapy could initiate an anti-tumor immune response by recruiting TILs and re-educating pro-tumoral M2 tumor-associated macrophages (TAMs) to anti-tumoral M1 TAMs to reverse the immunosuppressive microenvironment at the tumor [34, 58, 59]. The above results confirm that SIDP NMs could induce local hyperthermia to ablate tumor cells and evoke immunity under laser irradiation. However, weak immune activation is insufficient to inhibit the growth and metastases of malignant tumors; combined immunotherapy is necessary.

**Figure 7.**
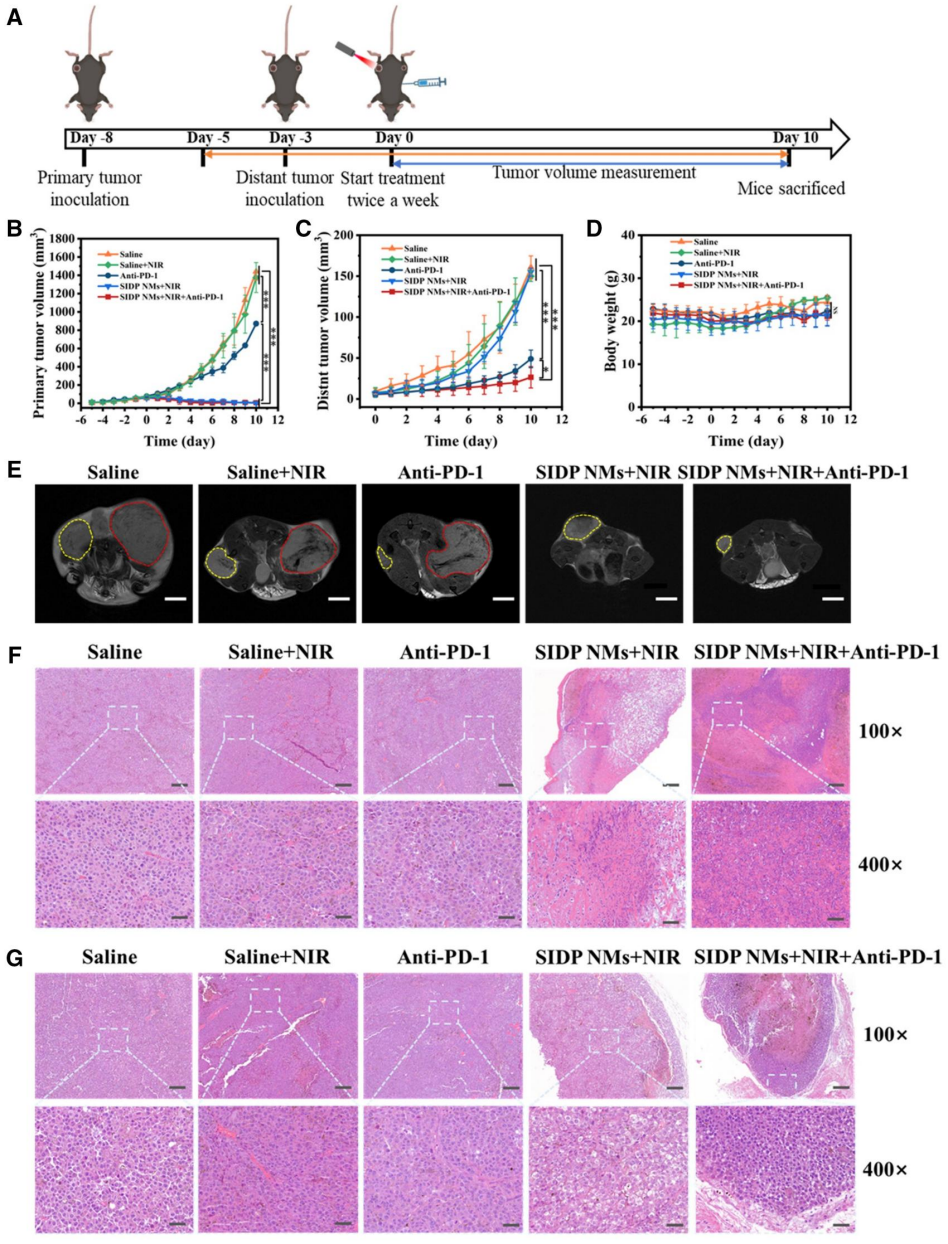
*In vivo* tumor suppressive effect of SIDP NMs on mouse melanoma-bearing mice. (**A**) Schematic diagram of the experimental design for *in vivo* treatment. (**B**) Primary and (**C**) distant tumor volume changes in melanoma-bearing mice before and after treatment with saline (i.v.), saline plus laser irradiation (i.v.), PD-1 inhibitor (i.p.), SIDP NMs plus laser irradiation (i.v.) and SIDP NMs plus laser irradiation combined with PD-1 inhibitor. (**D**) Changes in body weight of tumor-bearing mice before and after different treatments. (**E**) Representative MRI images of primary tumor (red) and distant tumor (yellow) at the end of therapy in different treatment groups; scale bar, 5 mm. H&E staining of (**F**) primary and (**G**) distant tumor after 10 days of treatment with saline, saline plus laser irradiation, anti-PD-1 treatment, SIDP NMs plus laser irradiation and anti-PD-1 treatment plus SIDP NMs plus laser irradiation; scale bar, 200 μm (upper) and 50 μm (lower).

Based on the desirable photothermal conversion capacity of SIDP NMs and MRI-guided *in vivo*, PPT/PDT combined immunotherapy was conducted according to the treatment schedule shown in Figure 7A. As shown in the primary tumor growth graph in Figure 7B, the primary tumors exhibited significant tumor growth inhibition after treatment with SIDP NMs + NIR and anti-PD-1 + SIDP NMs + NIR groups for 10 days, while the tumors grew rapidly in the saline and saline + NIR groups. Although PTT and PDT have been attracting much attention for their superior local therapeutic effects, the application of PTT and PDT was still restricted by the inability to suppress metastases. Thus, immunotherapy, along with its systemic features, was the ‘ideal companion’ for PTT/PDT [7, 60, 61]. To investigate the therapeutic effects of PTT/PDT combined immunotherapy on primary tumors and metastases, the therapeutic effects of smaller distant tumors after primary tumor implantation for 5 days were used to simulate the study of the efficacy of photothermal/photodynamic co-immunotherapy on melanoma metastases. Figure 7C presents the growth curves of distant tumors treated with saline, saline + NIR, SIDP NMs + NIR, anti-PD-1 and SIDP NMs + NIR + anti-PD-1, respectively. Anti-PD-1 therapy alone had a satisfactory inhibition efficacy for distant tumors, but the inhibition of primary tumors was inconspicuous. Contrary, although PTT and PDT theoretically had a mild immunostimulatory effect, it was weaker for distant tumors, so the growth of distant tumors was not regressed in the SIDP NMs + NIR group at all. In comparison, SIDP NMs + NIR in synergy with anti-PD-1 immunotherapy exhibited a superior ablative effect on primary tumors and an equal inhibition on distant tumors. Meanwhile, the weight of mice was always maintained after different treatments, indicating negligible side effects of the treatment modality (Figure 7D). Additionally, the therapeutic effects of representative mice were photographed during treatment (Supplementary Figure S18), and an MRI of the largest cross-section of tumor was acquired at the end of treatment (Figure 7E). The above results confirm that PTT/PDT triggered by SIDP NMs was sufficient to ablate the primary tumor *in vivo*, and combination with anti-PD-1 immunotherapy exhibited a significant distant tumor growth inhibition.

To further clarify the therapeutic effect of different treatment modalities on tumor-bearing mice, the mice were sacrificed at the end of treatment, and the tumors were peeled out and photographed (Supplementary Figure S19). Obviously, SIDP NMs + NIR had a significant ablative effect on the primary tumor, but the PTT/PDT-induced immune-activating effect hardly affected distant tumors. Only anti-PD-1 therapy had some inhibitory effect on smaller distant tumor but was ineffective against primary tumors. In contrast, SIDP NMs + NIR and anti-PD-1 treatment synergistically resulted in primary tumor ablation and distant tumor growth suppression, demonstrating a satisfactory therapeutic effect. Tumor tissues from each group were treated with H&E staining to observe changes at the cellular level. The large nuclear fragmentation of the primary tumor cells in SIDP NMs + NIR and SIDP NMs + NIR + Anti-PD-1 groups seen in Figure 7F illustrates the apoptosis and necrosis of the tumor cells. Regrettably, no obvious morphological changes were seen at the cellular level of the distant tumor, which was only inhibited by the immune activation triggered by anti-PD-1 and PTT/PDT (Figure 7I). Furthermore, to assess the safety of the treatment modality, the major organs of mice were stained by H&E at the end of the treatment (Supplementary Figure S20) and no morphological changes were observed in cells of the heart, liver, spleen, lung and kidney. These results confirmed the therapeutic efficacy and safety of SIDP NMs *in vivo* and their capacity to inhibit primary tumors and distant tumors in combination with PD-1 inhibitor.

**Figure 8.**
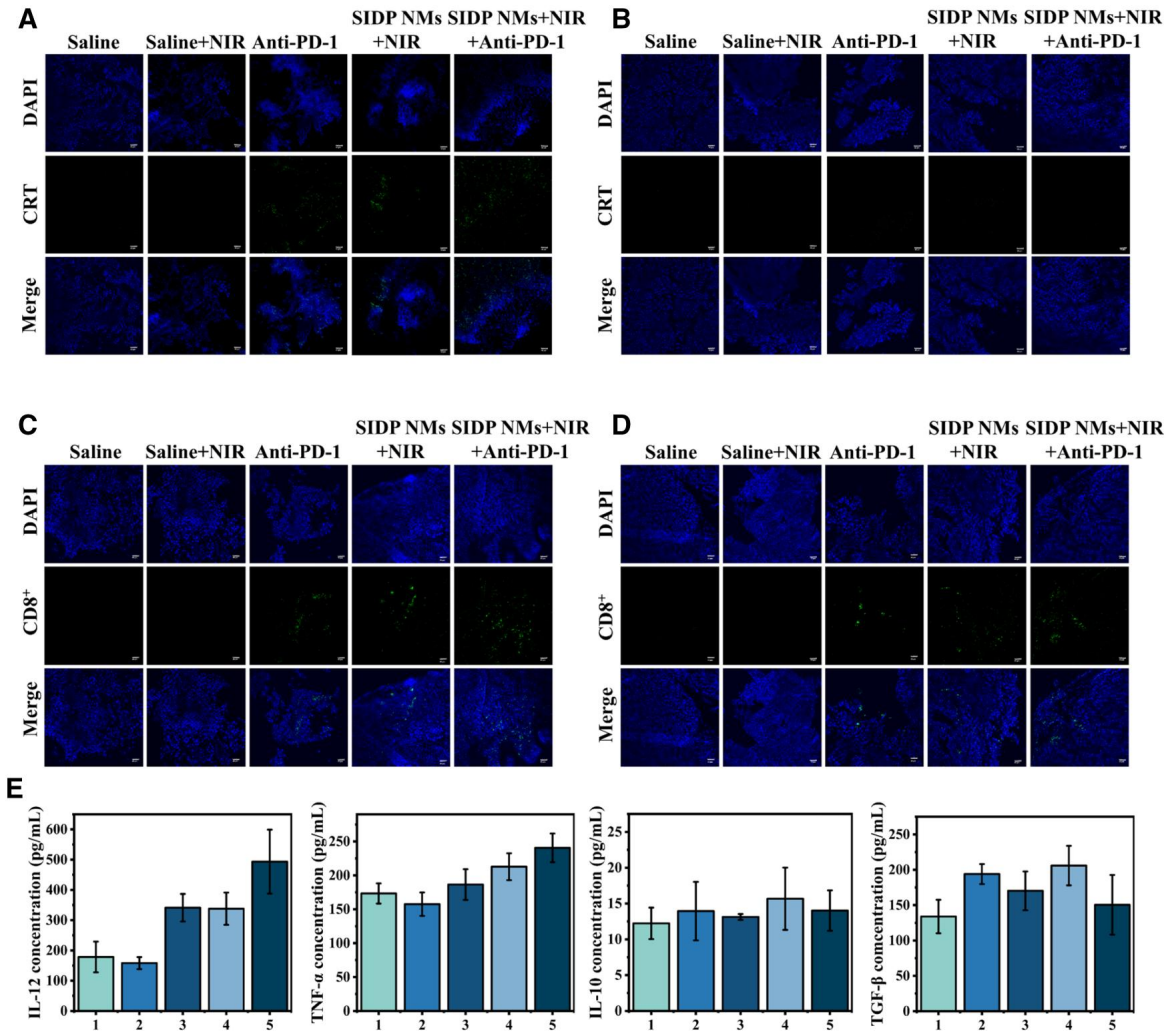
Effect of PTT/PDT combined with anti-PD-1 immunotherapy on T-lymphocyte and cytokines secretion. Representative immunofluorescence staining images of CRT (green) in (**A**) primary and (**B**) distant tumor tissues from different treatment groups (scale bar, 40 μm). representative immunofluorescence staining images of CD8^+^ (green) in (**C**) primary and (**D**) distant tumor tissues from different treatment groups (scale bar, 40 μm). (**E**) Effects of PTT/PDT combined with anti-PD-1 immunotherapy on cytokines secretion. 1: saline group, 2: saline plus laser irradiation group, 3: Anti-PD-1 treatment group, 4: SIDP NMs + NIR group, 5: SIDP NMs + NIR + Anti-PD-1 group (*n* = 3).

Immunogenic cell death is the transition of tumor cells to immunogenicity with the secretion of DAMP such as CRT, adenosine triphosphate and heat shock protein 70. Among them, CRT exposure acts as an ‘eat me’ signal to induce phagocytosis of antigen-presenting cells (APCs), which activates antigen presentation by APCs and further induces the enrichment of cytotoxic T-lymphocytes (CD8^+^ T cells) to exert anti-tumor effects [62, 63]. To clarify the immune activation of PTT/PDT, we investigated the CRT expression and the CD8^+^ T-cell enrichment at the tumor location in different treatment groups. As shown in Figure 8A–D, high CRT expression was found at tumor locations in the SIDP NMs + NIR group and in the SIDP NMs + NIR + anti-PD-1 group, and correspondingly, high CD8^+^ expression at primary and distant tumors was also seen. Furthermore, the combination therapy showed enhanced CD8^+^ expression as compared to PTT/PDT and anti-PD-1 therapy alone. In addition, cytokines with functions such as regulating cell proliferation, differentiation and migration, maintaining the stability of the internal environment and stimulating the inflammatory response influence the prognosis of tumors [64]. Therefore, cytokines were examined in mice from different treatment groups, and as shown in result Figure 8E, the levels of pro-inflammatory and anticancer cytokines IL-12 and TNG-α were significantly higher in the combination treatment group, whereas the anti-inflammatory and pro-tumor IL-10 and TGF-β remained relatively unchanged. These results confirm the immune-activating effect of PTT/PDT and show enhanced immune activation in combination with PD-1 inhibitor, overcoming the disadvantages of localized PTT/PDT and the ineffectiveness of immune checkpoint blockade.

## Conclusion

In summary, multifunctional nanomicelles were successfully constructed to serve as a therapeutic agent with MRI-guided PTT/PDT and explored the potential for primary tumor ablation and distant tumor eradication in combination with PD-1 inhibitor. Dopamine multivalent grafting shell, overcoming the disadvantage of the uncontrollable synthetic structure of PDA, maintains the photothermal/photodynamic properties and instability of ICG. Furthermore, SIDP NMs exhibited strong contrast effects both *in vitro* and *in vivo* because of the presence of SPIO, which might guide further precise photothermal treatment. The MRI results of melanoma-bearing mice showed that the SIDP NMs accumulated most in the tumor area at 5 h after tail vein injection. The subsequent SIDP NMs-triggered PTT/PDT synergized with PD-1 inhibitor anti-melanoma research validated that primary tumor ablation and distant tumor eradication were feasible and effective. Briefly, we developed an effective MRI-guided PTT/PDT nanoplatform that overcame the drawbacks of inconsistent therapeutic effects of traditional photosensitizers, laid the foundation for individualized application of PTT/PDT, and combined with PD-1 inhibitor to construct a multimodal treatment system. We subsequently validated its potential for MRI-guided photothermal/photodynamic/immunological combination therapy, providing a new therapeutic strategy for clinical oncology treatment.

## Supplementary Material

rbae019_Supplementary_Data

## Data Availability

The datasets and materials supporting the conclusions of this article are included within the article and its supporting information.
