# Supplementary material for: MRI-guided photothermal/photodynamic immune activation combined with PD-1 inhibitor for the multimodal combination therapy of melanoma and metastases
Source: Regen Biomater. 2024 Mar 14;11:rbae019. doi: 10.1093/rb/rbae019 (PMC10960927; doi:10.1093/rb/rbae019)
Supplement: rbae019_Supplementary_Data [file rbae019_supplementary_data.docx]

# Supporting Information

**MRI-guided photothermal/photodynamic immune activation combined with PD-1 inhibitor for the multimodal combination therapy of melanoma and metastasis**

Changqiang Wu^a, #, *^，Wei Chen^a,#^, Shuang Yan^a^, Jie Zhong^a^, Liang Du^a^, Chenwu Yang^a^, Yu Pu^a^, Yang Li^b^, Jiafu Lin^c^, Mei Zeng^d^, Xiaoming Zhang^a,*^

^a^ Medical Imaging Key Laboratory of Sichuan Province and School of Medical Imaging, North Sichuan Medical College, Nanchong 637000, P. R. China

^b^ Department of Radiology, Affiliated Hospital of North Sichuan Medical College, Nanchong 637000, P. R. China

^c^ Antibiotics Research and Re-evaluation Key Laboratory of Sichuan Province, Sichuan Industrial Institute of Antibiotics, School of Pharmacy, Chengdu University, Chengdu 610106, P. R. China

^d^ Institute of Basic Medicine and Forensic Medicine, North Sichuan Medical College and Institute of Rheumatology and Immunology, The Affiliated Hospital of North Sichuan Medical College, Nanchong, 637000, P. R. China

^#^ The authors wish it to be known that, in their opinion, the first two authors should be regarded as joint First Authors

* Corresponding authors:

Changqiang Wu, Medical Imaging Key Laboratory of Sichuan Province and School of Medical Imaging, North Sichuan Medical College, Nanchong 637000, P. R. China

E-mail: wucq1984@nsmc.edu.cn

Telephone: +86-13990723836

Xiaoming Zhang, Medical Imaging Key Laboratory of Sichuan Province and School of Medical Imaging, North Sichuan Medical College, Nanchong 637000, P. R. China

E-mail: zhangxm@nsmc.edu.cn

Telephone: +86-13808271001

**Supplementary materials and methods**

**Synthesis of superparamagnetic iron oxide nanocrystal**

Oleic acid-coated superparamagnetic iron oxide nanocrystals were synthesized using the thermal decomposition method. Briefly, 1 mmol Fe(acac)_3_, 5 mmol 1,2-hexadecanediol, 3 mmol oleic acid, and 3 mmol oleylamine were mixed in a double-necked round-bottom flask. The reaction was stirred at condensation reflux until the temperature gradually increased to 200°C under nitrogen protection and benzyl ether as the solvent, and maintained for 2 h. Then, gradually increase the system temperature to 300°C and maintain for 1 h. The reaction mixture was cooled to room temperature by removing the heat source, then the product was washed twice by centrifugation at 5000 rpm for 10 min with the addition of anhydrous ethanol. Finally, hexane was added to improve the dispersion of the nanoparticles.

**Synthesis of dopamine, oleylamine, and polyethylene glycol grafted polyaspartic acid**

Dopamine, oleylamine, and polyethylene glycol grafted polyaspartic acid (PAsp-*g*-DA/OAm/PEG) polymers were synthesized in three steps: polysuccinimide (PSI) and methoxy polyethylene glycol amino group (Mw 2000 Da) was dissolved in dimethyl sulfoxide and reacted at 80°C for 48 h under nitrogen atmosphere. Under nitrogen protection, oleylamine was added and reacted at 80°C for another 24 h. Then, dopamine hydrochloride and triethylamine were dissolved in dimethylsulfoxide and added to the reaction system at 80°C for 24 h, followed by adding sodium hydroxide solution and continuing the reaction. Finally, the pH of the solution was adjusted to 6 with hydrochloric acid and dialyzed in ultrapure water for 24 h (dialysis bag molecular weight cutoff 10 kDa), the final product was collected and lyophilized.

**Characterization**

Nuclear magnetic resonance hydrogen spectroscopy (^1^H NMR, Bruker, 400 MHz, US) was used to demonstrate the successful synthesis of the amphiphilic grafted polymer PAsp-*g*-DA/OAm/PEG. The hydrodynamic diameter size and surface zeta potential of the nanomicelles were measured by dynamic light scattering (DLS, Malvern Zetasizer Nano ZS90, UK). Fourier transform infrared spectrometer (FT-IR, thermos scientific, Nicolet iS5, USA) was employed to measure chemical bonds in SIDP NMs. Transmission electron microscopy (TEM, Hitachi, HT7820, Japan) was used to characterize the morphologies and size of SIDP NMs. TEM images were collected at 120 kV after phosphotungstic acid staining. The absorption spectra were measured by a UV-Vis spectrophotometer (Shimazu, UV-1900i, Japan). The relaxation time and relaxation efficiency of SIDP NMs solutions were measured and calculated using 0.5 T (Niumag, PQ001-20-015V, China), 1.4 T (Bruker, Minispec mq60, USA), and 3.0 T (GE, discovery MR750, USA) MR instruments respectively.

**Encapsulation efficiency and loading capacity**

To investigate the encapsulation ability of nanomicelles, the SIDP NMs were lyophilized and re-dispersed in solution of methanol and chloroform, and the concentration of ICG was determined with quantitative analysis by UV-Vis spectrophotometer. The encapsulation efficiency (EE) and drug loading capacity (LC) of SIDP NMs were calculated by the following formulas:

$$EE=\frac{Mass of encapsulated ICG}{Total mass of added ICG}\times100\%$$

$$LC=\frac{Mass of encapsulated ICG}{Mass of nanomicelles}\times100\%$$

**In vitro photothermal properties evaluation**

To investigate the photothermal properties of SIDP NMs, water, Free ICG, PAsp-DA/OAm/PEG nanomicellar solutions and SIDP NMs with different Fe concentrations (25, 50, 75, 100 µg/mL) and were irradiated with 808 nm laser (1 W/cm^2^) for 10 min, and 75 µg/mL SIDP NMs solution irradiated with different power densities (1.0, 1.5, 2.0 W/cm^2^) were also investigated. The temperature was recorded by photographing with an infrared thermal imaging camera (Fotric226s, China) during irradiation.

To investigate the photothermal conversion efficiency of SIDP NMs, the infrared imaging camera was employed to record the temperature changes of 200 µL SIDP NMs and free ICG solution, when they were irradiated by 808 nm laser (1 W/cm^2^) and cooling. Under the same conditions, the temperature change of water was recorded to calculate the thermal losses of the solvent. Finally, the photothermal conversion efficiency of ICG and SIDP NMs was calculated by the following formulas:

$$\theta=\frac{T_{sur}-T}{T_{sur}-T_{max}} (1)$$

$$t=-\tau_{s}Ln\theta(2)$$

$$\tau_{s}=\frac{m_{d}C_{d}}{hs} (3)$$

$$Q_{dis}=hs\left( T-T_{sur} \right) (4)$$

$$\eta=\frac{hs\left( T_{max}-T_{sur} \right)-Q_{dis}}{I(1-{10}^{-A})} (5)$$

Where *θ* is the thermal driving temperature, *T_sur_* is the ambient temperature, *T_max_* is the maximum temperature of the solution, m_d_ is the mass of the sample, *C_d_* is the heat capacity of the solvent, *τ_s_* is the system time constant, *I* is the laser power density, *h* is the thermal transfer coefficient, *s* is the surface area of the container, *A* is the absorption of the solution at 808 nm.

For evaluating photothermal stability, the changes in the characteristic absorption of SIDP NMs and ICG solutions after being irradiated by 808 nm laser (1 W/cm^2^) for different times were monitored by UV-Vis spectrophotometer. Furthermore, the temperatures of SIDP NMs and free ICG solutions were recorded during four cycles of 808 nm laser (1 W/cm^2^) on/off.

**In vitro photodynamic performance assay**

1,4-diphenyl-2,3-benzofuran (DPBF), and 2′,7′-dichlorofluorescin diacetate (DCFH-DA) probes were employed to evaluate the photodynamic properties of SIDP NMs. DPBF probes were employed to evaluate the SIDP NMs-triggered singlet oxygen production. 10 μL of DPBF solution dissolved in DMSO (2 mg/mL) was added to 2 mL of SIDP NMs solution (10 µg/mL), and then irradiated by an 808 nm laser (1 W/cm^2^) for various time. Subsequently, characteristic absorption of DPBF at 417 nm was monitored using UV-Vis spectrophotometer. DCFH-DA probes were used to investigate intracellular SIDP NMs-triggered ROS production. B16-F10 cells (7×10^4^ cells) were planted in confocal microscopic dishes. SIDP NMs (Fe concentration: 10 µg/mL) were added and incubated for 4 h under a 5% CO_2_ atmosphere at 37°C, meanwhile, the positive group was stimulated with rosup. Then, DCFH-DA (10 mM) dispersed in RPM-1640 medium was added and incubated for 20 min. After discarding the excess probe, the cells were irradiated by 808 nm laser (1 W/cm^2^) for 5 min. Finally, fluorescent images were obtained using a confocal microscope (Olympus, FV3000， Japan) equipped with 488 nm excitation light.

**Table S1.** *T*_1_and *T*_2_ relaxivity of SIDP NMs with different component ratios under different main magnetic fields.


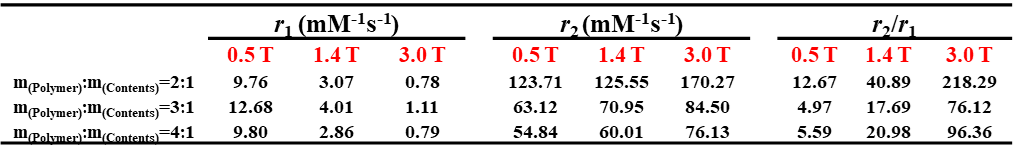


^a^ maintain m_(SPIO)_:m_(ICG)_ = 2:1


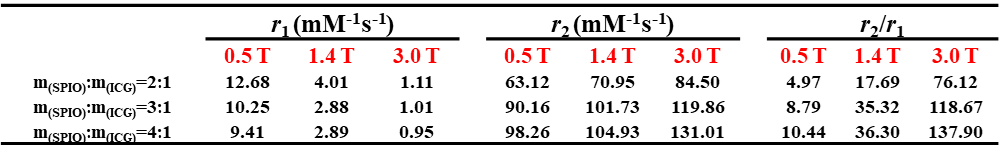


^b^ maintain m_(Polymer)_:m_(Contents)_ = 3:1


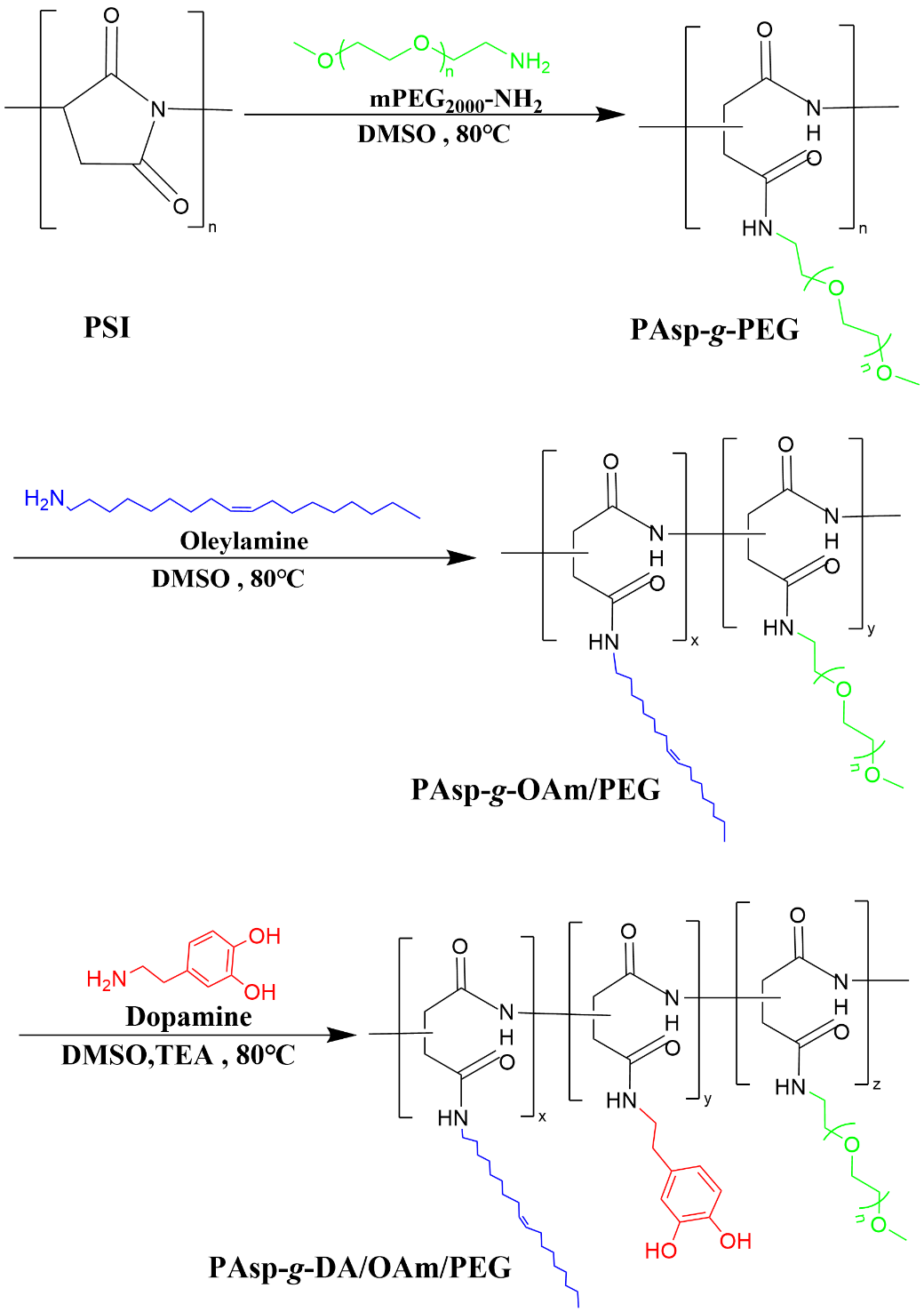


**Fig. S1** Synthesis pathway of amphiphilic graft polymer PAsp-*g*-DA/OAm/PEG.


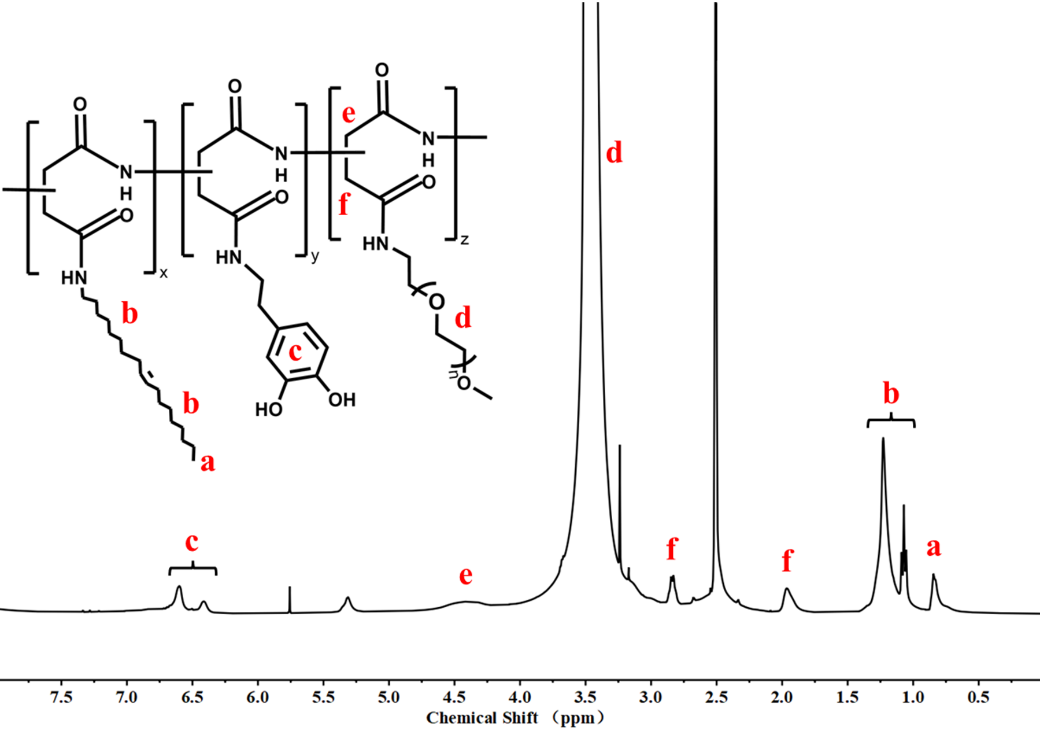


**Fig. S2** ^1^H NMR spectrum of the graft polymer PAsp-*g*-DA/OAm/PEG.


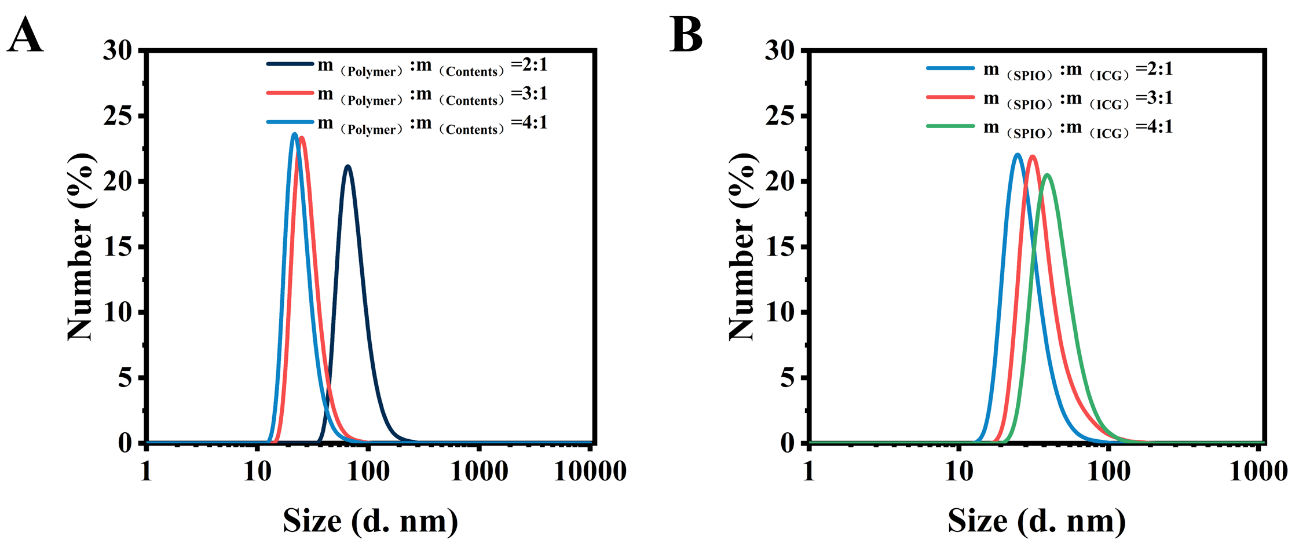


**Fig. S3** Size distribution of SIDP NMs with different component ratios measured by DLS. Maintain m_(SPIO)_:m_(ICG)_ = 2:1 when exploring the ratio of polymer to contents; m_(Polymer)_:m_(Contents)_ = 3:1 when exploring the ratio of SPIO to ICG.


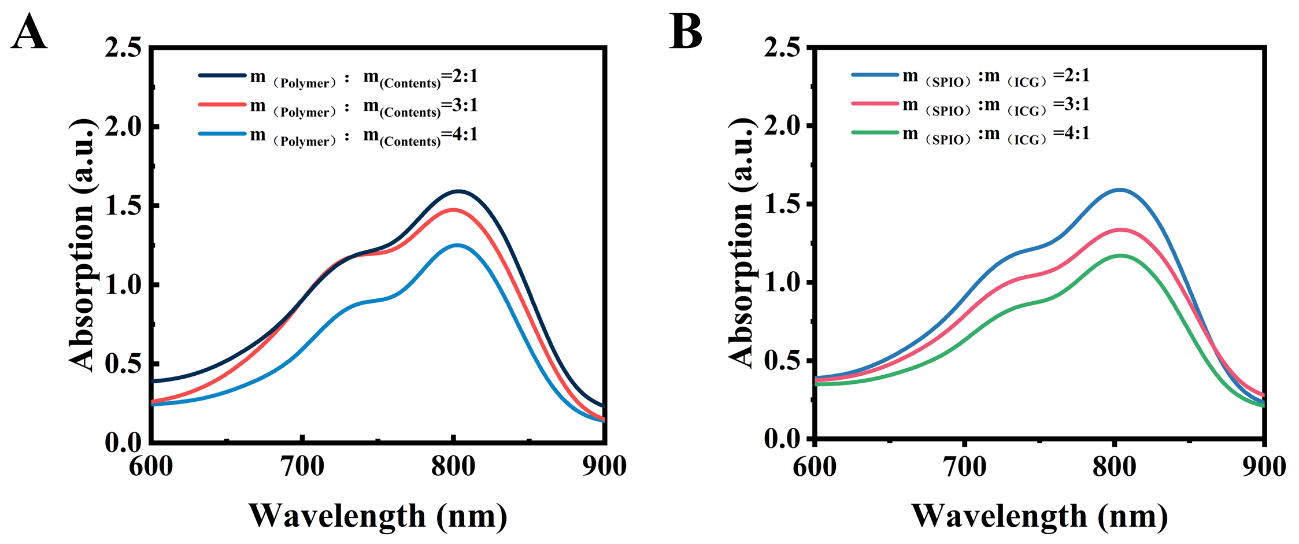


**Fig. S4** UV-Vis absorption spectra of SIDP NMs with different component ratios. Maintain m_(SPIO)_:m_(ICG)_ = 2:1 when exploring the ratio of polymer to contents; m_(Polymer)_:m_(Contents)_ = 3:1 when exploring the ratio of SPIO to ICG.


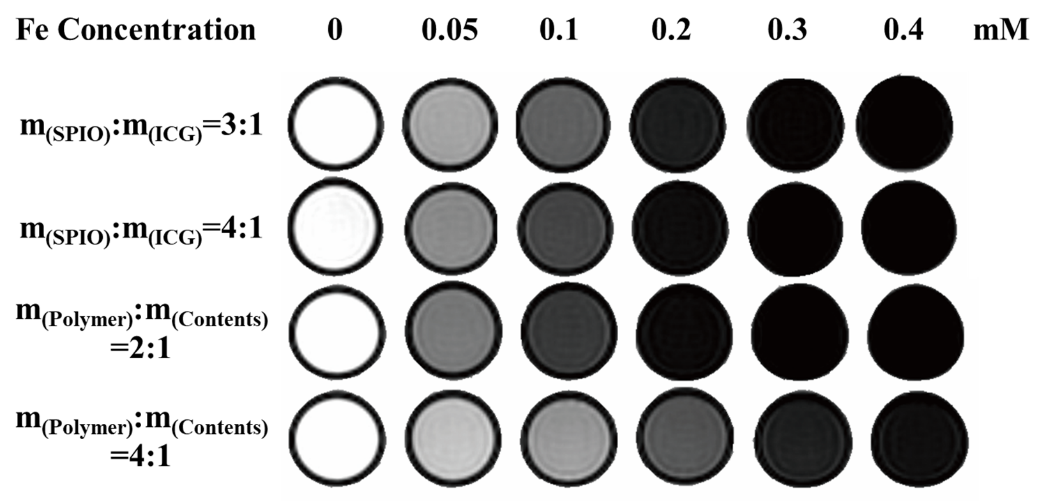


**Fig. S5** *T*_2_-weighted MRI images of SIDP NMs with different composition ratios at different iron concentrations in clinical 3.0 T MRI devices (FSE sequence, TR = 3500 ms, TE = 130 ms). Maintain m_(SPIO)_:m_(ICG)_ = 2:1 when exploring the ratio of polymer to contents; m_(Polymer)_:m_(Contents)_ = 3:1 when exploring the ratio of SPIO to ICG.


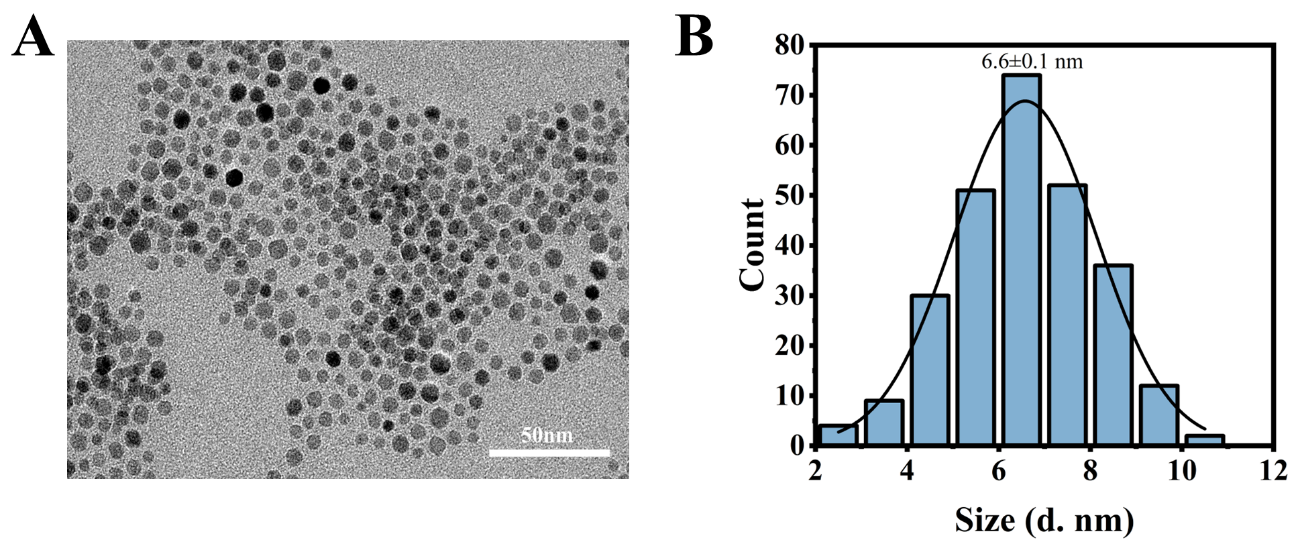


**Fig. S6** (A) TEM image of organic phase SPIO. (B) Particle size distribution is measured by (A).


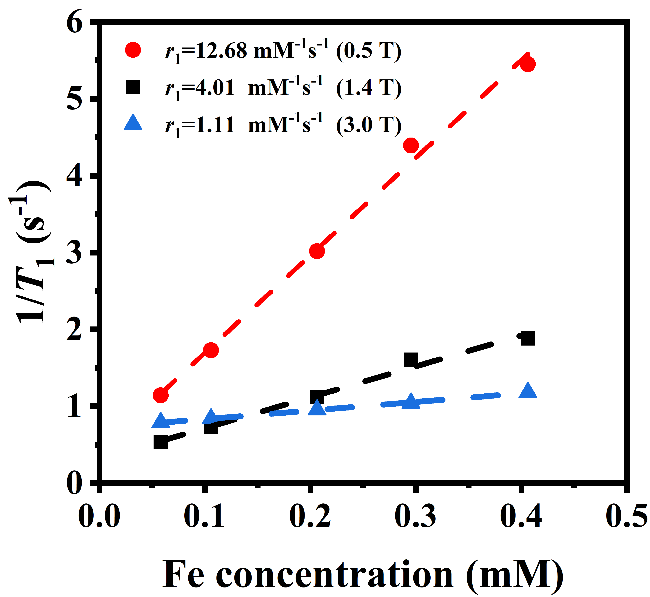


**Fig. S7** *T*_1_ relaxation efficiency-iron concentration curve of SIDP NMs solution under 0.5 T, 1.4 T, and 3.0 T main magnetic fields respectively.


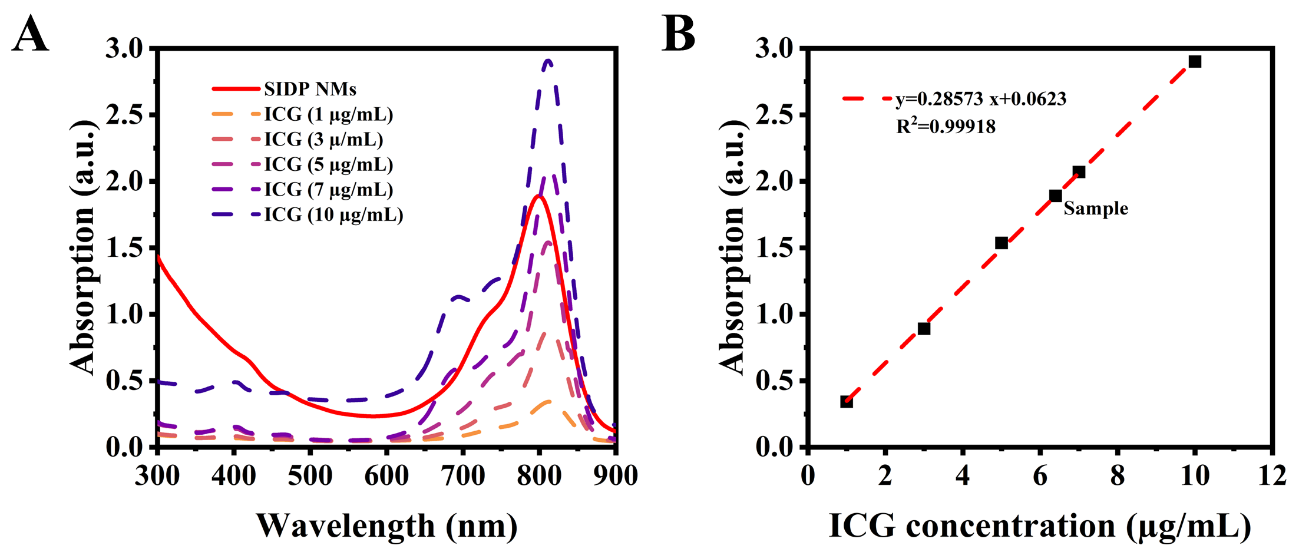


**Fig. S8** Measurement of ICG content in SIDP NMs by quantitative UV-Vis absorption spectroscopy.


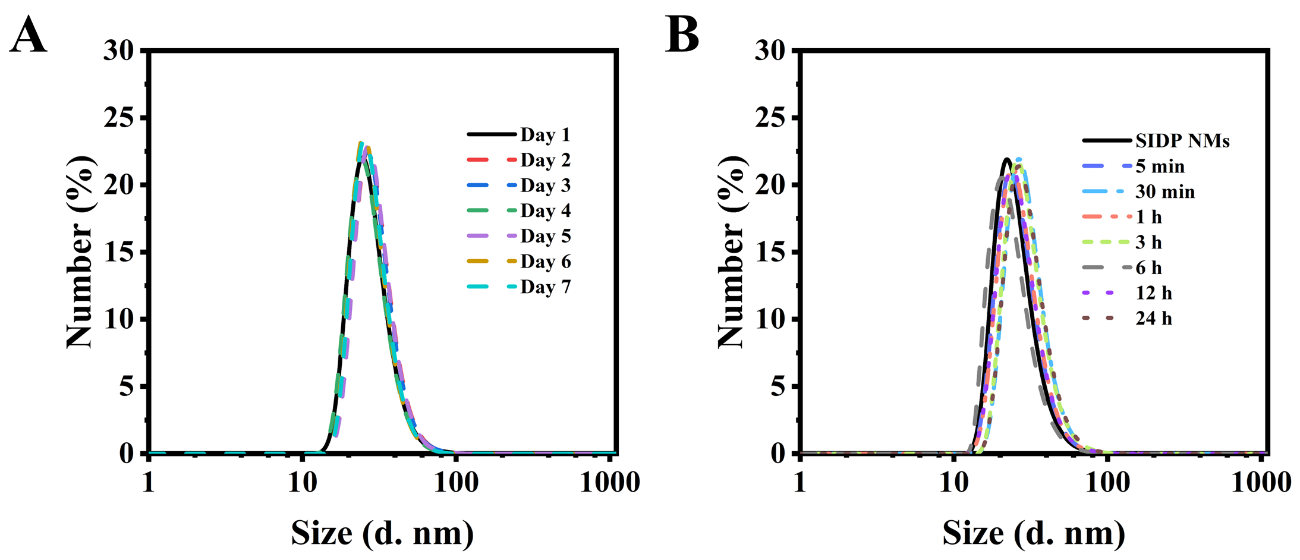


**Fig. S9** (A) Hydrodynamic diameter changes of SIDP NMs over 7 days. (B) Size variation of SIDP NMs incubated with 20% (v/v) FBS for 24 h.


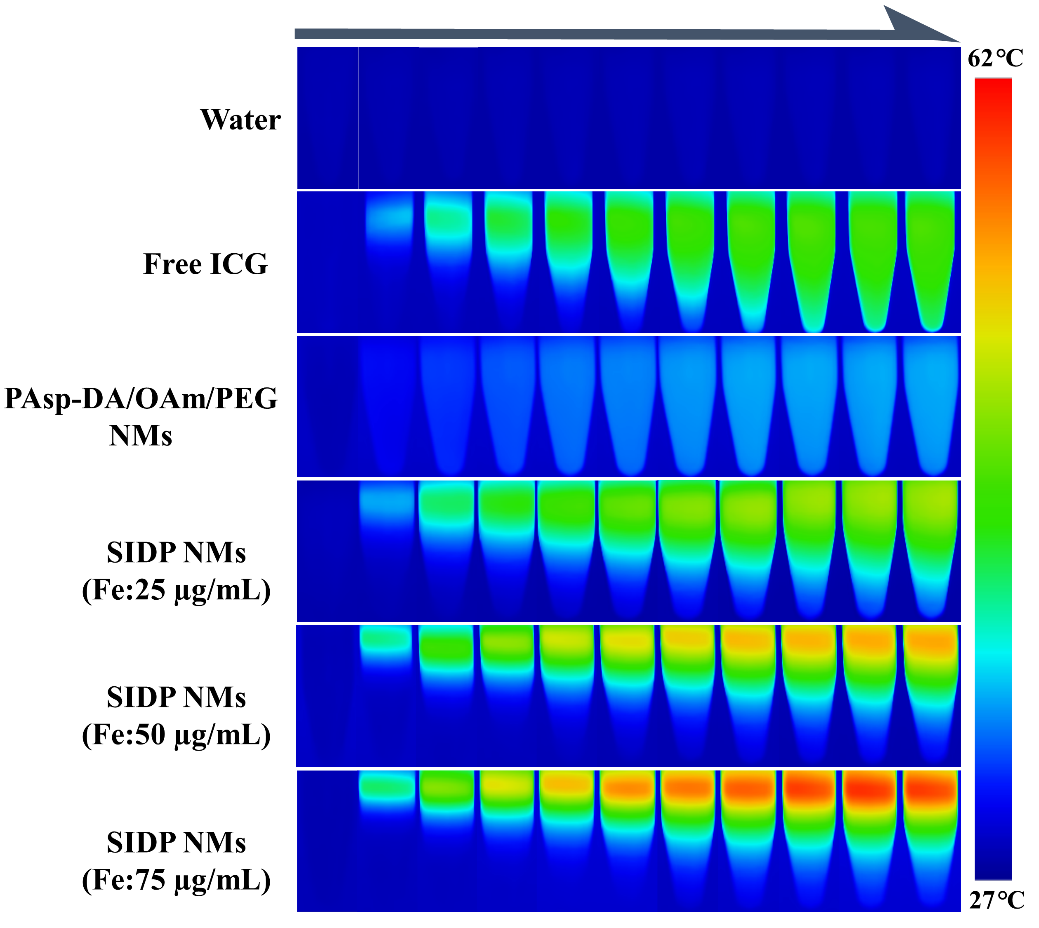


**Fig. S10** Infrared thermal imaging pictures of SIDP NMs aqueous solution with different concentrations, PAsp-DA/OAm/PEG NMs, ICG, and water under 808nm laser irradiation (1 W/cm^2^).


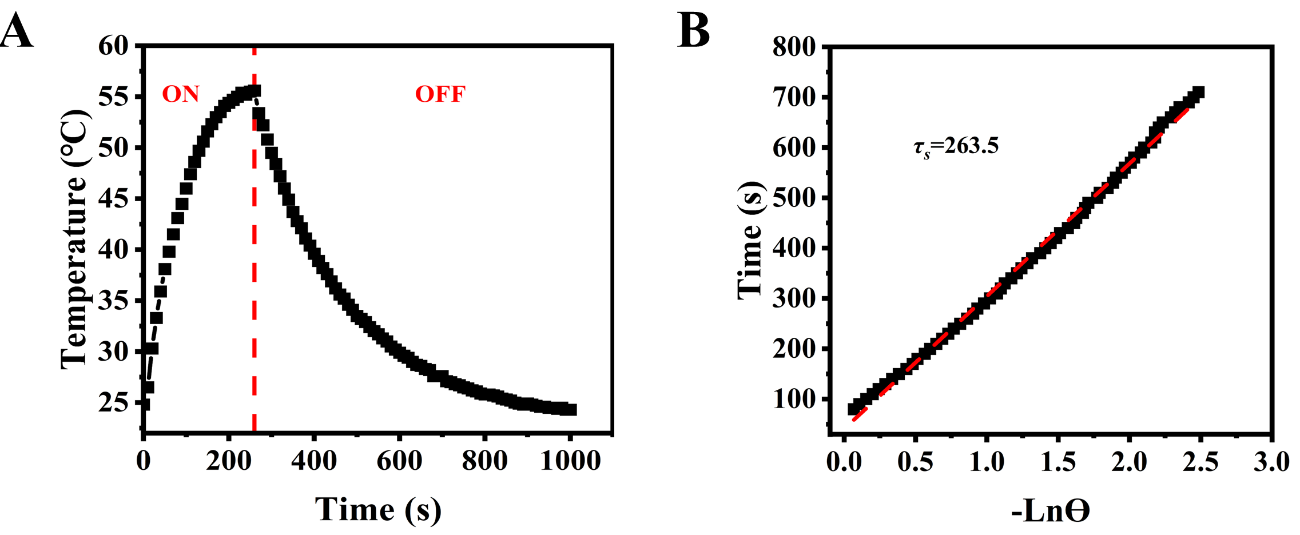


**Fig. S11** (A) Photothermic heating curves of ICG solution under 808 nm irradiation (1 W/cm^2^) for 260 s followed by cooling to room temperature. (B) Linear correlation of the cooling times versus negative natural logarithm of driving force temperatures. The time constant is calculated as *τ_s_* = 263.5 s.


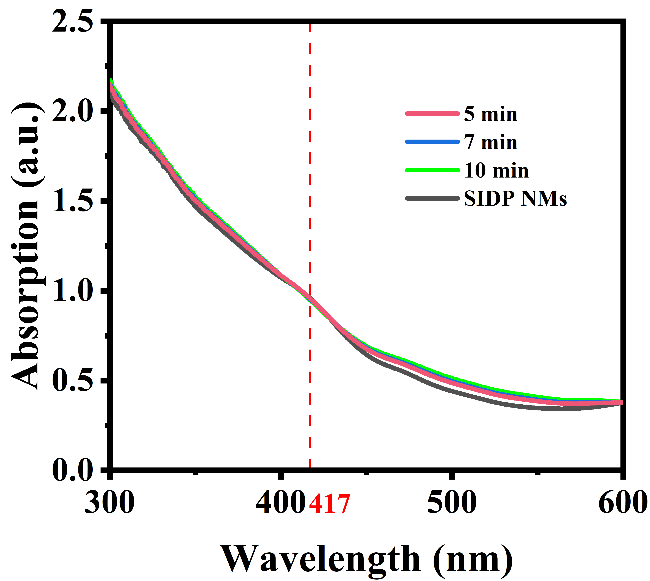


**Fig. S12** Absorption changes at 417 nm after 808 nm laser irradiation of SIDP NMs solution without DPBF for different time.


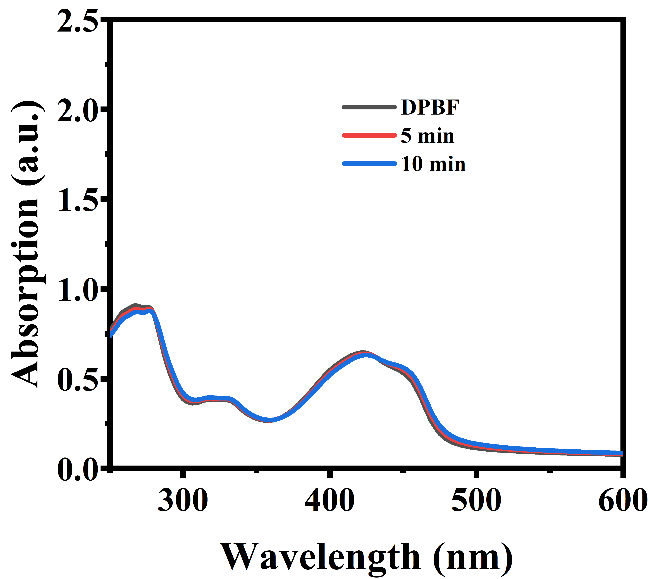


**Fig. S13** Absorption spectra of DPBF solution under 808 nm laser irradiation (1 W/cm^2^) for different time.


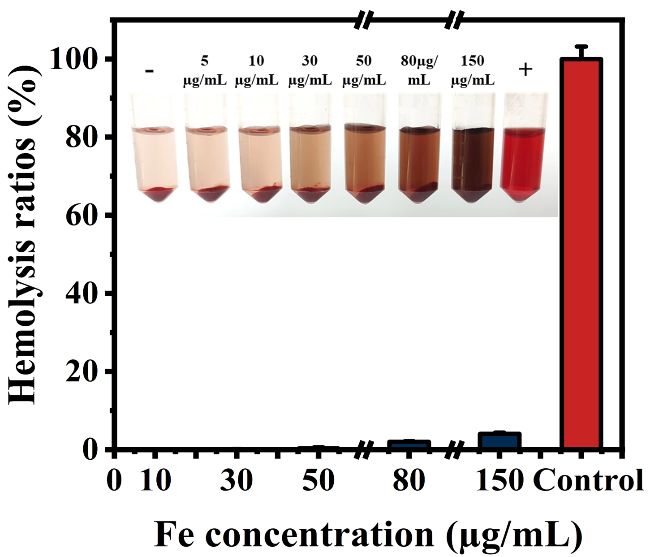


**Fig. S14** Hemolysis rates and photographs after co-incubation of erythrocytes and different concentrations of SIDP NMs solution for 3 h. PBS was used as a negative control and 0.1% Triton X-100 as a positive control.


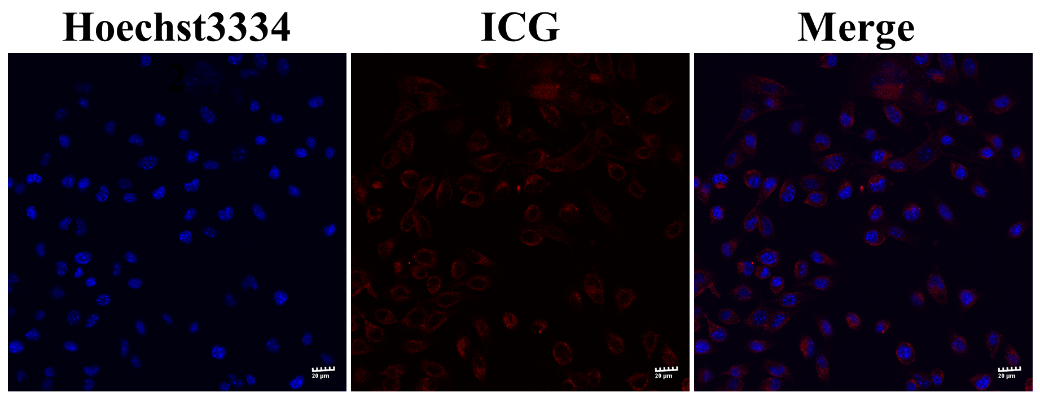


**Fig. S15** Fluorescence imaging of B16-F10 cells stained with Hoechst 33342 after cultured with SIDP NMs for 6 h; scale bar, 20 μm.


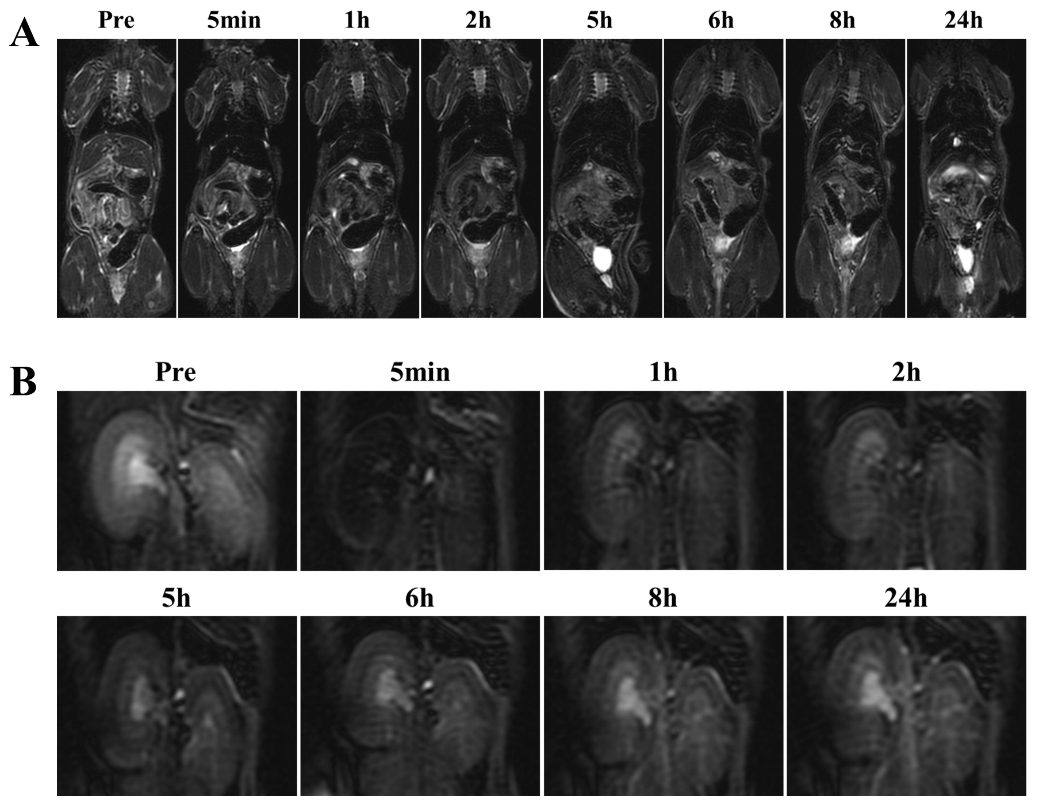


**Fig. S16** In vivo *T*_2_-weighted imaging of the (A) liver and (B) kidney at different time points after intravenous injection of SIDP NMs (5 mg/kg).


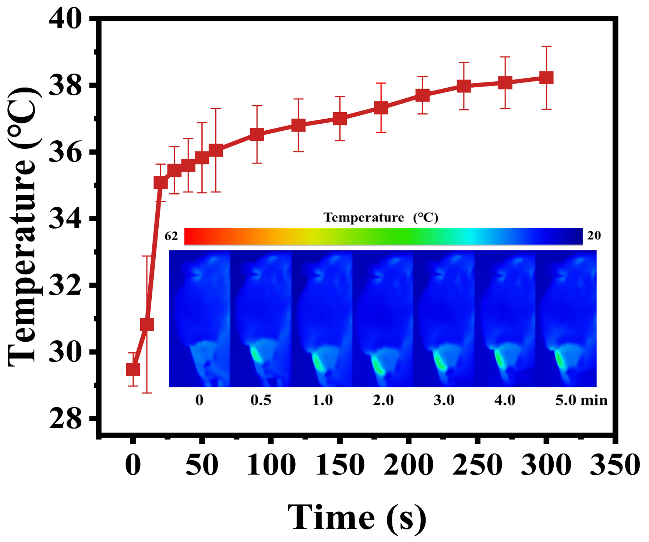


**Fig. S17** Infrared thermography and temperature change curves of the mouse skin within 5 min under 808 nm laser irradiation (0.7 W/cm^2^).


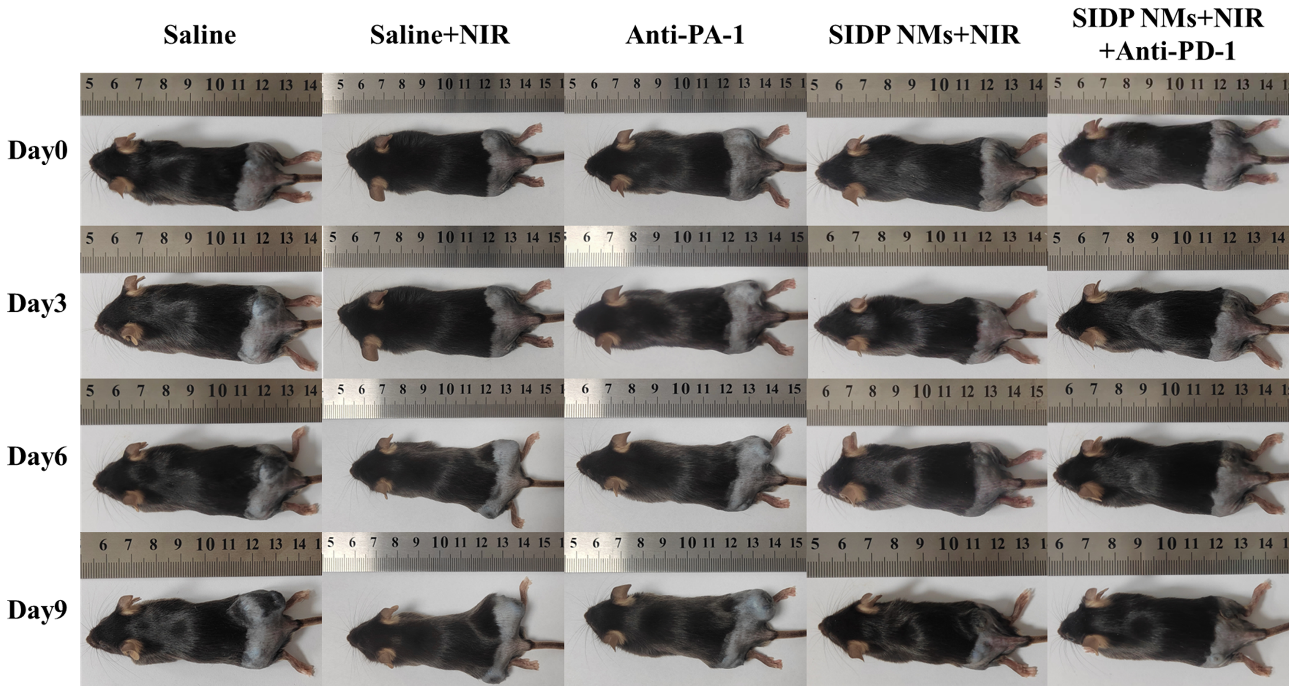


**Fig. S18** Representative photographs of mice during treatment in the saline group (i.v.), saline plus laser irradiation group (i.v.), Anti-PD-1 treatment group (i.p.), SIDP NMs + NIR group (i.v.), and SIDP NMs + NIR + Anti-PD-1 group (i.v.).


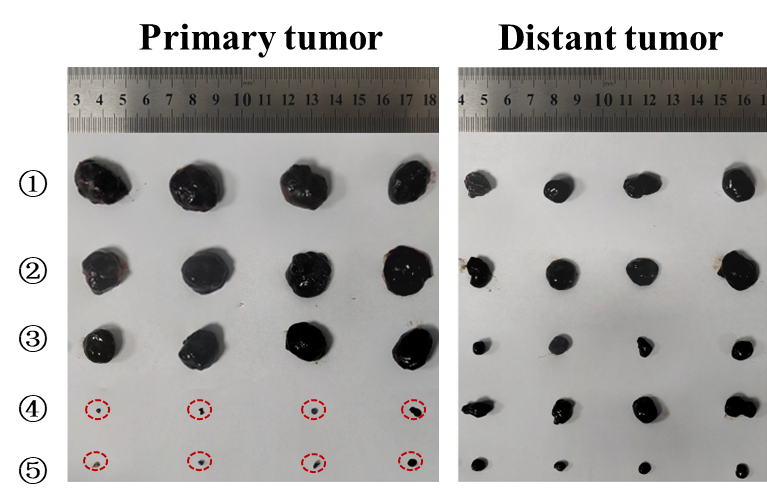


**Fig. S19** Primary and distant tumors stripped from mouse melanoma-bearing mice of different treatment groups at the end of treatment. ① Saline group (i.v.), ② Saline plus laser irradiation group (i.v.), ③ Anti-PD-1 treatment group (i.p.), ④ SIDP NMs + NIR group (i.v.), ⑤ SIDP NMs + NIR + Anti-PD-1 group.


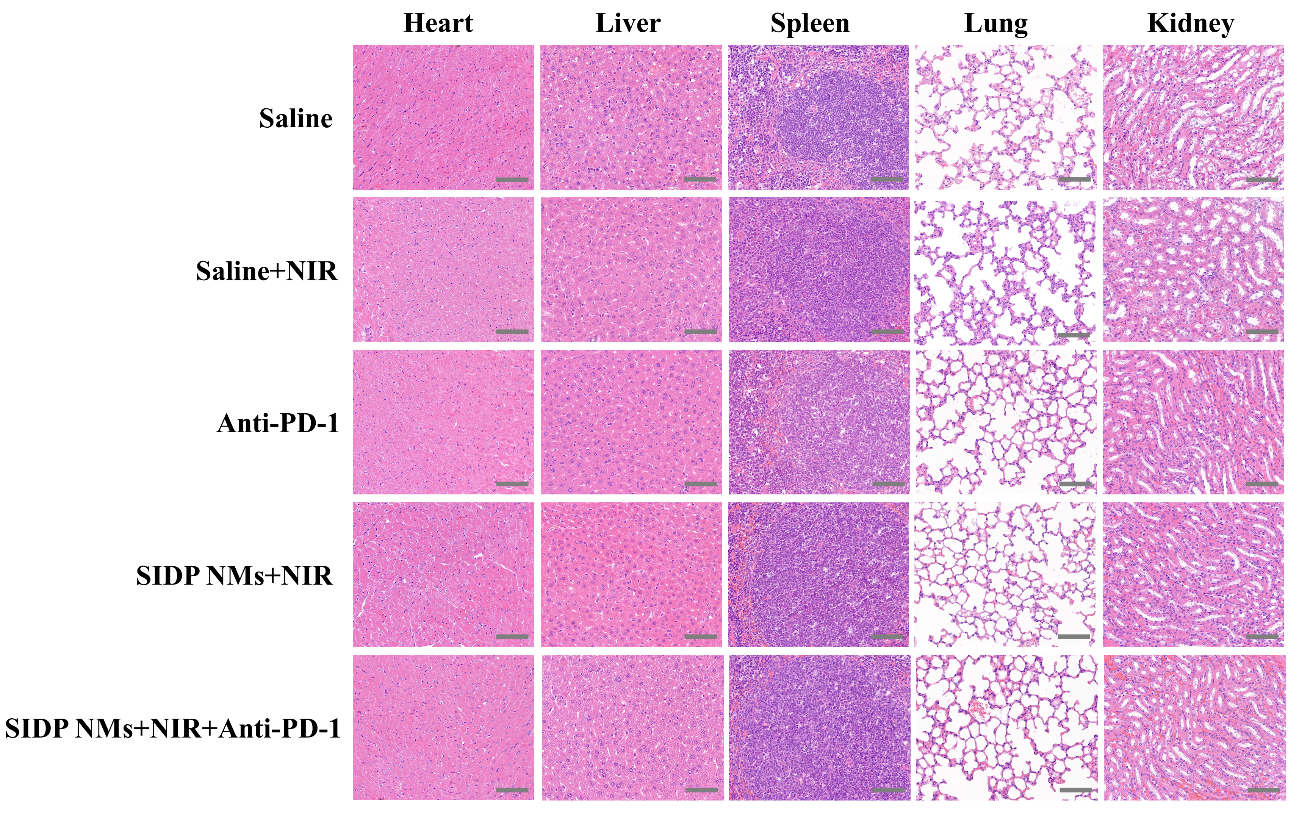


**Fig. S20** H&E staining of heart, liver, spleen, lung, and kidney tissue sections of mouse melanoma-bearing mice after 10 days of different treatments; scale bar, 80 μm.
